# The SoftWipe tool and benchmark for assessing coding standards adherence of scientific software

**DOI:** 10.1038/s41598-021-89495-8

**Published:** 2021-05-11

**Authors:** Adrian Zapletal, Dimitri Höhler, Carsten Sinz, Alexandros Stamatakis

**Affiliations:** 1 Computational Molecular Evolution group, Heidelberg Institute for Theoretical Studies, Heidelberg, Germany; 2 Institute for Theoretical Informatics, Karlsruhe Institute of Technology, Karlsruhe, Germany

**Keywords:** Bioinformatics, Software

## Abstract

Scientific software from all areas of scientific research is pivotal to obtaining novel insights. Yet the coding standards adherence of scientific software is rarely assessed, even though it might lead to incorrect scientific results in the worst case. Therefore, we have developed an open source tool and benchmark called SoftWipe, that provides a relative software coding standards adherence ranking of 48 computational tools from diverse research areas. SoftWipe can be used in the review process of software papers and to inform the scientific software selection process.

## Introduction

Scientific software (henceforth called software) has become pivotal for discoveries in almost all research fields, including emerging areas such as the digital humanities. The importance of software has become evident to the broad public by the plethora of computational tools (henceforth called tools) used to analyze SARS-CoV-2 data, for instance. However, due to the conditions under which software is typically being developed, such as, lack of sustainable funding for maintaining widely used tools, lack of time, and a lack of formal training in computer programming and software engineering, the software quality with respect to coding standards adherence (henceforth called software quality for brevity) exhibits a large variance. More importantly, software quality is typically not taken into account in the software development, software paper review, or in the tool selection process, for instance, when two computational tools provide analogous functionality.

The term ’coding standards adherence’ (or ’software quality’) is admittedly fuzzy, but offers a handle to perform largely automated analyses of scientific open source tools based on a set of objective criteria and measures. Such an automated analysis of numerous tools would be hard if one wanted to apply proper software verification techniques. When a tool exhibits ’bad’ software quality, this does absolutely not imply that it is erroneous. However, it does imply that the probability for it to generate faults that might, in the worst case, lead to paper retractions or incorrect results obtained with the faulty tool is higher (based on results from the area of empirical software engineering^1,2^). See the introduction of^3^ for a list of some high profile retractions and corrections because of software errors. For the above reasons, we strongly advocate that software quality should be assessed in the development, review, and tool selection process.

To this end, and based on our previous work^4^ where we manually assessed the software quality of widely used tools for evolutionary biology, we introduce SoftWipe, a largely automated open source tool and benchmark for assessing the software quality of open source tools written in C or C++. We envision that SoftWipe can be used by authors/developers (e.g., requiring them to provide their SoftWipe score with the submission), reviewers and researchers to integrate software quality into the review process and to raise awareness about software quality and engineering issues which are crucial for the success of computational science in general.

The SoftWipe score is a relative score over a number of software quality indicators (see further below and the supplement for a description of these indicators). The best-ranking tool per indicator receives a score of 10 while the worst ranking tool per indicator receives a score of 0. Subsequently, we compute the average unweighted score per tool over all per indicator scores to obtain a global software quality ranking. Prior to the global score calculation, the per indicator scores are corrected for outliers (see supplement for details).

This global relative score might change over time as more tools are being added to the benchmark and will therefore be difficult to reference. We have therefore also devised an absolute fixed global score (see supplement for mathematical details) that does not change when further tools are added to the benchmark and that can therefore easily be referenced.

We use the following software quality indicators (normalized by average values per 1000 lines of code (LoC)) to rate the tools: number of compiler, sanitizer, and static code analyzer warnings as generated by a variety of tools, number of assertions used, cyclomatic code complexity which is a software metric to quantify the complexity/modularity of a program, inconsistent or non-standard code formatting, and the degree of code duplication. Further, we approximate the overall fraction of test code by detecting test files and dividing the lines of test code by the overall lines of code. A file is considered a test file if the path or the file name contains the “test” keyword.

The selection of these quality indicators is based on results from the area of empirical software engineering and is thus not arbitrary. For instance, a study has established the connection between the amount of gcc compiler warnings and the revision numbers of source files in five industrial software projects^5^. Further, the predictive ability of the cyclomatic complexity as a metric for bug prediction has been demonstrated^6^. It has also been established that static analysis and code complexity metrics constitute early indicators of software defects^7^ and that code churn, complexity metrics, and static analysis results can be used to train an enhanced fault prediction model for embedded software^8^. The authors found that, when using a gradient boosting approach to conduct a simple binary classification of (automotive) software components into fault-prone and not fault-prone in a retrospective study, precision (number of fault-prone components returned divided by number of total components returned) and recall (number of fault-prone components returned divided by number of fault-prone components that should have been returned) values exceeded 90%.

Beyond these research results, coding recommendations by organizations that develop reliable or even security-critical software deploy analogous metrics and criteria. More specifically, NASA has developed risk-reducing coding rules, based on the analysis of previous incidents, with 4 distinct levels of code compliance. Their level 1 rule (“minimal standard of workmanship for all code”) mainly requires that “all code can pass both the compiler and a good static source code analyzer without triggering warnings” (see https://cacm.acm.org/magazines/2014/2/171689-mars-code/fulltext). Analogous approaches are being used at CERN. See, for instance, the Master thesis by Thomas Hofer, (https://infoscience.epfl.ch/record/153107/files/ESSCAT-report-for-printing.pdf) or the list of static source code analysis tools recommended for CERN developers at https://security.web.cern.ch/recommendations/en/code_tools.shtml. Finally, the INFER tool we use in SoftWipe is also being deployed by Facebook for code analysis and development (see https://research.fb.com/downloads/infer/).

To further justify the selected indicators we computed the Spearman rank correlation between individual per-indicator rankings and the overall SoftWipe ranking. For all individual indicators, except for clang-tidy and unique, there is either a moderate or a high degree of correlation with the overall SoftWipe score.

Another approach to establishing that our indicators are reasonable and meaningful is to correlate them with the amount of bug reports as documented in bugzilla or git-logs, for instance. However, tools for accurate automatic identification of true bugs in git-logs are difficult to develop and show 50% precision and 90% recall^9^. In the area of scientific software development, this becomes even more challenging, as some of the tools we studied do simply not have git-logs. Furthermore, unlike in industrial software development projects, scientific software developers use highly heterogeneous conventions (if any) when formulating git messages. To this end, we have conducted experiments using three non-scientific codes (sumo https://github.com/eclipse/sumo, llvm-openmp, and llvm-pstl) for which there exists an ample repertoire of bugzilla or git-log bug reports. Under the assumption that the reported issues containing the “bug” keyword are trustworthy we find that, sumo has a rate of 7.7 bugs per 1000 LoC (SoftWipe score: 3.7), llvm-openmp has a rate of 4.0 bugs per 1000 LoC (SoftWipe score: 5.2), and llvm-pstl has a rate of 0.8 bugs per 1000 LoC (SoftWipe score: 7.4). Hence, our experiments indicate that there is a tendency for bug reports to decrease with increasing SoftWipe score.

A more detailed description of the quality indicators including an extended rationale for the inclusion of each one is provided in the supplement and in the “Methods” Section.

The current benchmark comprises 48 tools from a wide range of scientific fields such as, evolutionary biology, astrophysics, epidemiological simulation, hypergraph analysis, deep learning applied to DNA data, antigen typing, protein data structure mining, SAT (satisfiability) solvers, etc. The benchmark results are provided in Table 1. Note again that absolute fixed SoftWipe scores do not change when new tools are included in the benchmark, while relative scores do change (see supplement for details). A comprehensive Table including all individual software quality indicators as well as descriptions of the respective tools (including references) is available online at https://github.com/adrianzap/softwipe/wiki/Code-Quality-Benchmark and in the supplement.

**Table 1:** Absolute fixed and relative SoftWipe scores of 48 computational tools from a wide range of scientific fields such as computer science, evolutionary biology, astrophysics, and bioinformatics.

Program name	Absolute score	Relative score
genesis-0.24.0	9.0	9.0
fastspar	8.2	8.4
parallel-STL	7.4	7.0
raxml-ng_v1.0.1	7.3	7.5
kahypar	7.3	7.8
ExpansionHunter-4.0.2	7.2	7.2
naf-1.1.0/unnaf	6.8	7.0
bindash-1.0	6.8	6.7
**dawg-1.2**	6.8	6.9
naf-1.1.0/ennaf	6.8	7.0
virulign-1.0.1	6.5	6.6
Treerecs-v1.2	6.5	6.8
glucose-3-drup	6.4	6.9
swarm-3.0.0	6.3	6.1
RepeatsCounter	6.3	6.2
samtools-1.11	6.0	6.5
bpp-4.3.8	5.9	6.3
ntEdit-1.2.3	5.7	6.1
prank-msa	5.5	5.9
IQ-TREE-2.0.6	5.5	5.6
dna-nn-0.1	5.3	5.1
openmp	5.2	5.4
ngsTools/ngsLD	5.2	4.9
emeraLD	5.2	5.3
defor	5.1	5.3
copmem-0.2	5.1	5.2
BGSA-1.0	5.0	5.4
phyml-3.3.20200621	4.9	5.3
dr_sasa_n	4.8	5.5
HLA-LA	4.8	5.6
SF2	4.6	4.9
**Seq-Gen-1.3.4**	4.6	4.9
clustal-omega-1.2.4	4.4	5.0
Gadget-2.0.7	4.4	4.6
cellcoal-1.0.0	4.4	4.2
minimap2-2.17	4.3	4.6
ms	4.3	4.6
MrBayes-3.2.7a	3.9	3.9
prequal	3.9	4.3
cryfa-18.06	3.8	4.1
vsearch-2.15.1	3.8	4.4
sumo	3.7	3.9
PopLDdecay	3.6	3.7
crisflash	3.5	4.3
athena-public-version-21.0	3.4	3.7
mafft-7.475	3.1	3.1
covid-sim-0.13.0	2.7	3.0
**INDELibleV1.03**	1.9	2.3

The three DNA sequence simulation tools dawg, seq-gen, and indelible that have similar functionality are highlighted in bold font.

One important observation is that the three out of the five top scoring tools (genesis-0.24.0, raxml-ng_v1.0.1, kahypar) have been developed by researchers with formal training in pure computer science. In addition, parallel-STL is a compiler and toolchain project that was initiated by the computer science department of the University of Illinois. Further, the 6 worst scoring tools have all been developed by self-taught programmers without formal training in computer science.

For an example of how the benchmark can be used, let us consider the three evolutionary molecular sequence data simulators dawg^10^, seq-gen^11^, and indelible^12^ (highlighted in bold font in Table 1). These tools implement highly similar functionalities and models. Our benchmark suggests that dawg, is less likely to fail, albeit it may of course, still contain programming errors while indelible might not, despite the inferior code quality. The indelible tool, for instance, would not pass the NASA level 1 compliance test.

It is also noteworthy that the developers of three (genesis-0.24.0, raxml-ng_v1.0.1, kahypar) out of the top five tools had access to a pre-release version of SoftWipe as they are members or collaborators of our lab and improved their tools to obtain higher SoftWipe scores. This highlights the utility of SoftWipe within a lab such as ours that predominantly develops computational tools, as it fosters a healthy competition among lab members to develop the best-scoring tool. In fact, the regular deployment and integration of SoftWipe in the development process has also helped to avoid a bug in at least one occasion: We detected a missing break statement in a C++ switch instruction, which caused an overestimation of the so-called speciation rate in our GeneRax tool^13^. This bug would not have been noticed otherwise, as it did not induce any crash or implausible results. Thus, SoftWipe can also aid the developer to guide code refactoring and develop code that will be easier to maintain and extend.

Nonetheless, the importance of implementing thorough tests for developing reliable software needs to be emphasized. SoftWipe takes this into consideration in two ways. First, by taking into account the most rudimentary form of tests as implemented via assertions. This is also required by NASA level 1; a study by Casalnuovo et al.^14^ suggests, that functions *with* assertions *do* have fewer defects. Second, by taking into account the fraction of test code present in the overall code.

Given the current SARS-CoV-2 pandemic it is also worth noting that the CovidSim tool^15^ obtains the second worst score among all 48 tools tested.

We are well aware of the fact that SoftWipe is likely to generate controversial debates, as software quality does neither induce lack of code correctness, nor an erroneous underlying scientific model. Nonetheless, in the current absence of any standard routines for assessing scientific software quality and based upon the results of empirical software engineering research that establishes a correlation between software quality and failure, SoftWipe represents a step forward, as it is easy to use and does not require a substantial investment of time. This does not only apply to the software submission and review process, but also to software development. SoftWipe can also help to objectify controversial discussions about software quality, as was the case with the CovidSim tool (see e.g., https://www.imperial.ac.uk/news/197875/codecheck-confirms-reproducibility-covid-19-model-results). We are therefore convinced that a routine deployment of SoftWipe or analogous tools at various stages of the software development and publication process can substantially contribute to improving software quality across all scientific fields, raise general awareness about the importance of software quality in the respective research communities, and reduce the number of program faults.

Regarding future developments of SoftWipe, we intend to also include valgrind (https://www.valgrind.org/) in future releases of SoftWipe despite the fact that it is substantially slower than Infer. While Infer is fast, we have observed that it frequently fails to execute on some of the tools. We will also implement version control for the code analysis tools that are being used by SoftWipe as versions 11 and 12 of clang-tidy, for instance, tend to report more warnings than version 10.

## Methods

### SoftWipe overview

SoftWipe is a pipeline, written in Python3, that uses predominantly freely available static and dynamic code analyzers to assess the code quality of software written in C/C++. SoftWipe initially compiles the software using the clang compiler (https://clang.llvm.org) and subsequently executes the software with *clang-sanitizers*. To accomplish this, the user is required to provide a text file with a command line call of the specific software such that it can be executed. For our benchmark, we used minimal toy examples as provided by the authors of the software, if available. In cases, where no examples were available, we set them up to the best of our abilities using freely available public datasets.

Subsequently, SoftWipe counts the lines of code, excluding comments and empty lines, and executes the static code analyzers described in the subsequent Subsection. Using the output of the static code analyzers, SoftWipe computes a score for each static analyzer and then outputs an overall score. Note that, strictly speaking, the clang-sanitizers are not static, but dynamic analyzers. SoftWipe retains *all* intermediate code analysis results such that software developers can access code quality analysis results in one single place and analyze as well as resolve potential issues. Note that SoftWipe, does not further extend or summarize the output of the analysis tools for refactoring purposes. If one or several of the code analysis tools fail to produce a score, SoftWipe will exclude the respective category from the overall score calculation. The user can also exclude analysis tools from the overall score. This can be done via a respective command line switch (e.g., –exclude-infer to exclude the Infer analysis; see supplement for further details).

### Description of and rationales for indicators

Static analysis techniques come in a wide variety of flavors, ranging from light-weight style checkers to full-blown formal verification tools. The latter are used in areas where high levels of software reliability are mandatory. They suffer, however, from long or even prohibitive runtime requirements. In addition, substantial manual effort is typically required to apply them and to interpret their results.

In SoftWipe we therefore predominantly use light-weight static analysis tools, as well as additional techniques based on source code metrics:

#### Compiler warnings and sanitizers

We compile each tool using the clang C/C++ compiler and count the number of warnings. We chose clang, as we empirically found that it typically produces more warnings than other compilers such as gcc. We activate almost all warnings (118 in total) for this. We have manually assigned a weight (severity level) of 1, 2, or 3 to each warning type. The weight assignment process is described in the supplement. A warning with a weight of 3 is considered as being most severe; for instance, implicit type conversions that might result in precision loss constitute weight 3 warnings. We calculate a weighted sum of these warnings, where each warning issued contributes its weight (1, 2, or 3) to the weighted sum. Additionally, we execute the tool with clang sanitizers (ASan and UBSan). If the sanitizers issue warnings, we add them to the weighted sum. Sanitizer warning severeness defaults to a weight of 3. Since ASan and UBSan perform dynamic (run-time) analyses of programs, we run them using examples as provided by the respective developers. If no examples are available, we set them up to the best of our abilities using freely available public datasets. ASan, the AddressSanitizer, detects memory-related errors such as out-of-bound heap or stack accesses, or use-after-free errors. UBSan, the UndefinedBehaviorSanitizer, detects program statements that result in undefined behavior according to the C/C++ language standard. The compiler and sanitizer score is calculated as a rate corresponding to the weighted sum of warnings per total LoC.

#### Assertions

We count the number of assertions (C-style assert(), static_assert(), or custom assert macros, if defined) per total LoC.

#### Cppcheck

This tool focuses on detecting undefined behaviour and dangerous coding constructs. It categorizes its warnings; we have thus assigned a weight to each category, analogous to the compiler warnings. The cppcheck score is calculated as the weighted sum of warnings per total LoC.

#### Clang-tidy

Similar to cppcheck, clang-tidy is a static analyzer for detecting typical programming errors like style violations or interface misuse. The clang-tidy tool categorizes its warnings; we have assigned each category a weight, analogous to the cppcheck and compiler warnings. The clang-tidy score is calculated as the rate of the weighted sum of warnings found per total LoC.

#### Cyclomatic complexity

The cyclomatic complexity is a software metric to quantify the complexity/modularity of a program. We use the lizard tool to assess the cyclomatic complexity of our benchmark tools. Lizard reports the number of functions that are considered too complex, relative to the total number of functions. Lizard considers a function as “too complex” if its cyclomatic complexity, its length, or its parameter count exceeds a certain threshold value. The score is calculated using the cyclomatic complexity value returned by lizard.

#### Unique rate

We determine the amount of “unique code”; a higher amount of code duplication decreases this value. A low unique rate is problematic, as it may induce copy-paste errors. The score used here is simply the unique rate returned by lizard.

#### KWStyle

This is a configurable, light-weight style checker for C/C++. We configure KWStyle using the KWStyle.xml file that ships with SoftWipe. The KWStyle score is calculated as the rate of the number of warnings found by the static style analyzer KWStyle per total LoC.

#### Infer

This is a static analyzer for C/C++ and Objective-C developed by Facebook. It can detect null pointer dereferences, memory leaks, violations of coding conventions, or unavailable APIs. The Infer score corresponds to the rate of the the weighted sum of warnings per LoC.

#### Test count

SoftWipe also tries to detect files containing unit tests and calculates the test count rate as the fraction of LoC in test files divided by the overall number of LoC.

### SoftWipe code quality

We also assessed the quality of our SoftWipe code by deploying the following commonly used static analyzers for Python:

- Pylint (https://www.pylint.org/)
- Pyflakes (https://github.com/PyCQA/pyflakes)
- Radon (https://radon.readthedocs.io/en/latest/)

Overall, we found that the quality of SoftWipe is “good” (see supplement for details and a full transcript of the analyses).

The open source code of SoftWipe including the up-to-date benchmark and a user manual is available at: https://github.com/adrianzap/softwipe/wiki.

## Supplementary Information

Supplementary Information.

## References

[1] Briand, L. C., Wust, J., Ikonomovski, S. V., & Lounis, H. Investigating quality factors in object-oriented designs: an industrial case study. In *Proceedings of the 1999 International Conference on Software Engineering (IEEE Cat. No.99CB37002)* 345–354 (1999).

[2] Casalnuovo, C., Devanbu, P., Oliveira, A., Filkov, V., & Ray, B. Assert use in github projects. In *2015 IEEE/ACM 37th IEEE International Conference on Software Engineering* **1**, 755–766 (2015).

[3] Wilson G (2014). Best practices for scientific computing. PLoS Biol..

[4] Darriba D, Flouri T, Stamatakis A (2018). The state of software for evolutionary biology. Mol. Biol. Evol..

[5] Moser R, Russo B, Succi G (2007). Empirical analysis on the correlation between GCC compiler warnings and revision numbers of source files in five industrial software projects. Empir. Softw. Eng..

[6] Ferzund J, Ahsan SN, Wotawa F (2008). Analysing bug prediction capabilities of static code metrics in open source software. Software Process and Product Measurement.

[7] Omri S, Montag P, Sinz C (2018). Static analysis and code complexity metrics as early indicators of software defects. J. Softw. Eng. Appl..

[8] Omri, S., Sinz, C., & Montag, P. An enhanced fault prediction model for embedded software based on code churn, complexity metrics, and static analysis results. *ICSEA 2019* 189 (2019).

[9] Tian, Y., Lawall, J., & Lo, D. Identifying linux bug fixing patches. In *2012 34th international conference on software engineering (ICSE)* 386–396. IEEE (2012).

[10] Cartwright RA (2005). Dna assembly with gaps (dawg): Simulating sequence evolution. Bioinformatics.

[11] Rambaut A, Grass NC (1997). Seq-gen: An application for the Monte Carlo simulation of DNA sequence evolution along phylogenetic trees. Bioinformatics.

[12] Fletcher W, Yang Z (2009). Indelible: A flexible simulator of biological sequence evolution. Mol. Biol. Evolution.

[13] Morel B, Kozlov AM, Stamatakis A, Szöllősi GJ (2020). Generax: A tool for species-tree-aware maximum likelihood-based gene family tree inference under gene duplication, transfer, and loss. Mol. Biol. Evol..

[14] Casalnuovo, C., Devanbu, P., Oliveira, A., Filkov, V., & Ray, B. Assert use in github projects. In *Proceedings of the 37th International Conference on Software Engineering: Volume 1*, ICSE ’15, 755–766. IEEE Press. ISBN 9781479919345 (2015).

[15] Walker Patrick GT, Whittaker Charles, Watson Oliver J, Baguelin Marc, Winskill Peter, Hamlet Arran, Djafaara Bimandra A, Cucunubá Zulma, Mesa Daniela Olivera, Green Will (2020). The impact of covid-19 and strategies for mitigation and suppression in low-and middle-income countries. Science.

