# Supplementary material for: The SoftWipe tool and benchmark for assessing coding standards adherence of scientific software
Source: Sci Rep. 2021 May 11;11:10015. doi: 10.1038/s41598-021-89495-8 (PMC8113446; doi:10.1038/s41598-021-89495-8)
Supplement: Supplementary file 1 — Supplementary Information. [file 41598_2021_89495_MOESM1_ESM.pdf]

Supplement: The SoftWipe tool and benchmark for assessing coding standards adherence of scientific software

Adrian Zapletal[1,2], Dimitri Höhler[1,2], Carsten Sinz[2], and Alexandros Stamatakis[1,2]

[1]Computational Molecular Evolution group, Heidelberg Institute for Theoretical Studies, Heidelberg, Germany
[2]Institute for Theoretical Informatics, Karlsruhe Institute of Technology, Karlsruhe, Germany

April 16, 2021

1 Analysis tools

1.1 Compiler and sanitizer

`SoftWipe` compiles the scientific software in our benchmark with the `clang` compiler with a large set of enabled warning flags. That is, it uses `-Weverything` with the exception of the warnings listed below which we chose to exclude as we considered them as being excessively pedantic.

- -Wno-padded
- -Wno-c++98-compat
- -Wno-c++98-pedantic
- -Wno-c++11-extensions
- -Wno-c99-compat
- -Wno-newline-eof
- -Wno-source-uses-openmp

We classify the warnings subjectively into three categories: *could-fix*, *should-fix* and *must-fix* weighted by 1, 2, and 3, respectively. Here a higher weight implies a higher severity (e.g., implicit type conversions that may result in a

loss of precision). The weight assignments for warnings are shown in Table 1. The classification was conducted via an open discussion in the slack channel of our lab that currently comprises 8 members that all have formal training in pure computer science. While such a classification is indeed subjective, its application does not substantially change the ranking of the tools with respect to an unweighted warnings ranking of the tools (Spearman rank correlation: 0.979).

As already mentioned, we also execute the compiled executable with `clang` sanitizers (`AdressSanitizer` and `UndefinedBehaviorSanitizer`).

We employ the following sanitizer flags:

- -g
- -fno-omit-frame-pointer
- -fsanitize=address
- -fsanitize-recover=address
- -fsanitize=undefined

All sanitizer warnings receive a weight of 3 by default. We sum over the weighted warnings of the compiler and sanitizer and calculate the *compiler and sanitizer rate* as the weighted sum per total number of lines of code *(LOC)*.

warning	weight
-Wc99-extensions	1
-Wcast-qual	1
-Wchar-subscripts	1
-Wcomment	1
-Wcovered-switch-default	1
-Wdate-time	1
-Wdeprecated	1
-Wdeprecated-dynamic-exception-spec	1
-Wdeprecated-register	1
-Wdocumentation	1
-Wdocumentation-deprecated-sync	1
-Wdocumentation-unknown-command	1
-Wexit-time-destructors	1
-Wextra-semi	1
-Wglobal-constructors	1
-Wgnu-zero-variadic-macro-arguments	1
-Wmissing-declarations	1
-Wmissing-noreturn	1
-Wold-style-cast	1
-Woverloaded-virtual	1
-Wparentheses-equality	1
-Wreorder	1
-Wshadow-field	1
-Wshadow-field-in-constructor	1
-Wshadow-field-in-constructor-modified	1
-Wunknown-pragmas	1
-Wunreachable-code	1
-Wunused-exception-parameter	1
-Wunused-function	1
-Wunused-macros	1
-Wunused-template	1
-Wvarargs	1
-Wweak-vtables	1
-Wwritable-strings	1
-Wc11-extensions	2
-Wcast-align	2
-Wcomma	2
-Wdeprecated-declarations	2
-Wdouble-promotion	2
-Wempty-body	2
-Wexpansion-to-defined	2
-Wfor-loop-analysis	2
-Wformat	2
-Wformat-extra-args	2

-Wformat-nonliteral	2
-Wgnu-binary-literal	2
-Wimplicit-int	2
-Winvalid-source-encoding	2
-Wlanguage-extension-token	2
-Wlogical-not-parentheses	2
-Wlogical-op-parentheses	2
-Wmacro-redefined	2
-Wmissing-prototypes	2
-Wmissing-variable-declarations	2
-Wnested-anon-types	2
-Wnonnull	2
-Wparentheses	2
-Wpedantic	2
-Wreserved-id-macro	2
-Wshadow	2
-Wstrict-prototypes	2
-Wswitch-bool	2
-Wswitch-enum	2
-Wundefined-func-template	2
-Wunreachable-code-break	2
-Wunreachable-code-loop-increment	2
-Wunreachable-code-return	2
-Wunused-parameter	2
-Wunused-private-field	2
-Wunused-value	2
-Wunused-variable	2
-Wvexing-parse	2
-Wvla	2
-Wvla-extension	2
-Wzero-as-null-pointer-constant	2
-Wabsolute-value	3
-Wbad-function-cast	3
-Wconditional-uninitialized	3
-Wconstant-conversion	3
-Wconversion	3
-Wdelete-non-virtual-dtor	3
-Wfloat-conversion	3
-Wfloat-equal	3
-Wformat-security	3
-Wheader-hygiene	3
-Wimplicit-fallthrough	3
-Winfinite-recursion	3
-Wliteral-conversion	3
-Wmultichar	3
-Wnon-virtual-dtor	3

-Wnull-arithmetic	3
-Wnull-conversion	3
-Woverlength-strings	3
-Wpointer-bool-conversion	3
-Wpointer-sign	3
-Wreturn-type	3
-Wself-assign	3
-Wself-assign-field	3
-Wself-assign-overloaded	3
-Wself-move	3
-Wshift-sign-overflow	3
-Wshorten-64-to-32	3
-Wsign-compare	3
-Wsign-conversion	3
-Wsometimes-uninitialized	3
-Wstatic-self-init	3
-Wstring-plus-int	3
-Wstring-compare	3
-Wstring-conversion	3
-Wtautological-constant-compare	3
-Wtautological-pointer-compare	3
-Wtautological-type-limit-compare	3
-Wtautological-unsigned-zero-compare	3
-Wundef	3
-Wuninitialized	3
-Wvector-conversion	3
-Wabsolute-value	3
-Wbad-function-cast	3

Table 1: Compiler warning weights

1.2 Assertions

Assertions provide a means to ensure that conditions for a correct execution of a program are met and that a rudimentary from of testing is implemented. Thus, we consider a software containing more assertions as being of higher quality. `SoftWipe` outputs the *assertion rate* as the number of assertions (C-style `assert()`, `static_assert()`, or custom assert macros, if available) per total LOC. If the analyzed code contains custom assert macros, the user can specify them via regular expression (regex) using the following `SoftWipe` option `-a custom_assert_regex`.

1.3 Cppcheck

`Cppcheck` (http://cppcheck.sourceforge.net) is a further static code analysis tool that detects undefined behavior and potentially dangerous coding constructs.

warning	weight
information	0
style	1
performance	1
portability	3
error	3
warning	3

Table 2: Cppcheck warning weights

We weight the warnings according to the weightings presented in Table 2 and output a *cppcheck rate* of the weighted warnings per total LOC.

1.4 Lizard

`Lizard` (https://github.com/terryyin/lizard) is a cyclomatic complexity analyzer that outputs three metrics.

The first output is the 'cyclomatic complexity', which is a software metric to quantify the complexity/modularity of a program using the number of linearly independent paths in the control flow graph of the program. It assumes that intuitive complexity and graph-theoretic complexities correlate [1]. A linearly independent path is a path that has at least one unique edge that is not part of any other path. `Lizard` computes the cyclomatic complexity for each function, as well as the average cyclomatic complexity over all functions.

The second metric it calculates is the 'number of complex functions'. Lizard considers functions as being overly complex if their cyclomatic complexity, length, or parameter number exceeds a given threshold (i.e., 15 for cyclomatic complexity, 1000 for length, 100 for the number of parameters). The respective rate is calculated as the number of overly complex functions per total number of functions. As it is usually advisable to keep functions as short and simple as possible, these thresholds alert the programmer once they exceed them. The above thresholds of 1000 for the length and 100 for the number of parameters are the default values of lizard and are in principle arbitrary and debatable as is the case for any threshold parameter setting. Yet, based on common sense and programming experience, these default settings are comparatively liberal as we believe that most software engineers would consider them as being excessive. In other words, being liberal they only detect the worst cases of unstructured and overly complex code.

The third metric calculated by `Lizard` is the *unique rate* which outputs a score depending on the amount of duplicated code.

1.5 KWStyle

For larger software projects it is common to enforce coding guidelines to produce more readable and maintainable code.

**warning**	**weight**
performance	1
readability	1
boost	1
cpp-core-guidelines	1
misc	1
modernize	1
bugprone	2
clang-analyzer	2
mpi	2

Table 3: Clang-Tidy warning weights

The `KWStyle` tool (https://kitware.github.io/KWStyle/) automatically checks for adherence to certain common coding style guidelines. It also allows the programmer to set up a set of custom guidelines in a `KWStyle.xml` file. `KWStyle` then analyses the code, detects violations of these guidelines and outputs respective warnings. At present, `SoftWipe` employs a general `KWStyle.xml` file which limits the line length, requires spaces between operators, and prohibits multiple statements in the same line. We calculate a `KWStyle` score depending on the warnings generated per total LOC.

1.6 Clang-Tidy

`Clang-tidy` (https://clang.llvm.org/extra/clang-tidy/) is a static analysis tool which focuses on detecting typical programming errors, such as coding style violations or interface misuse which could lead to bugs. It generates several warning categories which we weight as shown in Table 3. We calculate the `Clang-tidy` rate as weighted warnings per LOC.

1.7 Infer

`Infer` (https://github.com/facebook/infer) also is a static analysis tool that can detect severe issues such as memory leaks. We therefore weight most of the warnings produced by `infer` with a weight of 3, as shown in Table 4. As before, we calculate the `infer` rate as weighted warnings per LOC.

1.8 Test Count

SoftWipe also tries to detect files containing unit tests (*test files*) and calculates the `test count` rate as the fraction of lines of code in test files divided by the overall lines of code. A file is considered a test file if the path or the name of the file contain the keyword “test”.

warning	weight
dead store	1
empty vector access	3
null dereference	3
memory leak	3
resource leak	3
uninitialized value	3

Table 4: Infer warning weights

2 Scoring

Each of the analysis methods listed above returns a *rate* which we use to calculate the intermediate score (one score for each method) which is in turn used to calculate the *overall score* as an unweighted average over all intermediate scores (see Equation 2.1, for overall score S_O, number of analyzers n and intermediate scores S_i). For the calculation of the intermediate scores we have two different approaches which we describe in the following. In order to make different analysis tools comparable, we calculate *worst* and *best* bounds from the corpus of tools that we have tested so far, excluding outliers. We call the rate r of a tool an outlier, if it lies outside Tukey's fences[2]. That is, for our set R_c, which contains all rates of programs in the benchmark for a category c, r is an outlier *iff* $r \notin [Q_1 - k(Q_3 - Q_1), Q_3 + k(Q_3 - Q_1)]$, for upper and lower quartiles Q_1, Q_3 of R_c and a non-negative constant k. We set $k := 1.5$, as proposed by Tukey. For analysis tools where a global best or worst bound exists (e.g., there can be no better case than observing 0 warnings), we explicitly set the bound to this value.

$$S_O = \frac{1}{n} \sum_{i}^{n} S_i \tag{2.1}$$

2.1 Relative scoring

We use relative scoring to compare a set of software programs with each other. To achieve this, we employ a linear formula scaled between the worst and best bound such that a rate x receives 0/10 if $x = worst$ and 10/10 of $x = best$. We update the worst and best bounds after each newly included software to the benchmark. Consequently, we have to update the relative scores in the benchmark every time we add new software. Hence, relative `SoftWipe` scores will not be stable over time as more software is being added and can not be easily referenced.

2.2 Absolute fixed scoring

The absolute fixed score is the score which the user receives as feedback including the intermediate and overall results. This absolute fixed score does not change over time with the addition of new software to the benchmark and can hence be referenced in submissions or publications. To achieve this, it does not suffice to simply fix the [*worst*, *best*] interval, since a rate x (see Section 1 for detailed definitions of the rates) of the new software being added can exceed that interval resulting in a score below 0 or over 10. Fixing the maximum/minimum scores for rates that exceed the interval yields distinguishing among these rates impossible. Thus, we employ the Sigmoid function $sig(x, x_0, k) = \frac{1}{1+e^{-k(x-x_0)}}$ as the scoring function and scale the parameters k and x using the *curve_fit()* function from the *scipy* library to produce a score between 0 and 10. We use the following code to scale the Sigmoid:

```
d = best - worst
x = rate - worst
thresh = 0.90
xval = [(1 - thresh) * d, 0.25 * d, 0.5 * d, 0.75 * d, thresh * d]
yval = [(1 - thresh), 0.25, 0.5, 0.75, thresh]
popt, pcov = curve_fit(sigmoid, xval, yval)
return 10 * sigmoid(x, *popt)
```

We define the return value of the function above as $sig'(x)$. To calculate the intermediate scores for every analysis method, we distinguish between three scenarios:

1. We cannot fix the *best* and *worst* boundaries a priori (e.g., cyclomatic complexity), see Equation 2.2.
2. We can fix the *best* boundary (e.g., compiler warnings), see Equation 2.3.
3. We can fix the *worst* boundary (e.g., number of assertions), see Equation 2.4.

$$S_i^1(x) = sig'(x) \tag{2.2}$$

$$S_i^2(x) = \begin{cases} sig'(x) & \text{if } x \geq \frac{best-worst}{2} \\ 10 \times \frac{x}{best-worst} & \text{else} \end{cases} \tag{2.3}$$

$$S_i^3(x) = \begin{cases} sig'(x) & \text{if } x \leq \frac{best-worst}{2} \\ 10 \times \frac{x}{best-worst} & \text{else} \end{cases} \tag{2.4}$$

We chose this specific implementation of the absolute score due to its high Spearman rank correlation of 0.972 to the relative score.

3 Executing SoftWipe

We developed and tested `SoftWipe` mainly on (and for) Linux-based systems. Thus the following recommendations may or may not apply to Windows or MacOS. `SoftWipe` requires `Python3` and the following python packages:

- numpy $\geq$ 1.17.4
- scipy $\geq$ 1.3.3

as well as the following tools:

- Clang (https://clang.llvm.org)
- Cppcheck (http://cppcheck.sourceforge.net)
- Clang-Tidy (part of LLVM tools http://llvm.org)
- Lizard (https://github.com/terryyin/lizard)
- KWStyle (https://kitware.github.io/KWStyle/)
- Infer (https://github.com/facebook/infer)

For Debian-based systems `SoftWipe` can try to download the required tools automatically.
`SoftWipe` can be executed using the following command:

```
softwipe.py [-c | -C] [-m | -M | -l target [target ...]]
            [-e executefile] programdir
```

Where
-c is for software written in C, **-C** for software written in C++
-m is for Make-based builds, **-M** for CMake-based builds, and **-l target** for builds where one needs to compile one or multiple target files directly.
The Makefile needs to use common variable names (e.g. ${CC} for the C compiler, ${CFLAGS} for the C compiler flags and ${LDFLAGS} for the linker flags) as `SoftWipe` uses them to inject its own compilers and flags.
-e specifies a file that contains the shell command required to run the tested software, for instance,

```
./executable -arg1 -arg2
```

programdir specifies the root of the tested software
Further `SoftWipe` options are displayed via the `--help` command.

4 Benchmark

We used `SoftWipe` to analyze 48 software tools written in C/C++. We selected 20 of those tools by scrutinizing recent *Bioinformatics Application Notes* papers. We did not include GUI applications, pipelines, and software that we did not manage to execute. We did not analyze external libraries used by the softwares as they are frequently larger than the actual newly written software. This might lead to a distortion of the overall score. On the other hand one would typically not expect an application developer to fix the issues of the external libraries they are using. Libraries also tend to fill a certain niche which in general makes it unlikely to find suitable alternatives for every use case. When available, we used the execution examples provided by the tested softwares for sanitizer execution. For tools which did not include examples, we prepared simple execution examples ourselves. The most recent results are available on the `SoftWipe github` page (https://github.com/adrianzap/softwipe/wiki/Code-Quality-Benchmark). These results may change in the future as new software might be added to the benchmark. The benchmark as it was at the time of the publication of this paper can be found in Sections 6.1 and 6.2. We split the benchmark into two tables: One containing absolute values used to calculate the scores and one containing the absolute scores for each category as well as the absolute and relative overall scores. 'N/A' (not available) entries indicate that `SoftWipe` could not produce a score for the specific category (e.g., because the respective analysis tool terminated with an error). `SoftWipe` automatically excludes such results from the overall score.

5 Quality of Softwipe Code

As already mentioned in the main text we assessed the quality of our `SoftWipe` code by deploying the following commonly used static analyzers for python:

- Pylint (https://www.pylint.org/)
- Pyflakes (https://github.com/PyCQA/pyflakes)
- Radon (https://radon.readthedocs.io/en/latest/)

5.1 Pylint

Pylint is a static analyzer that focuses on the adherence to Python's PEP 8 style guide (https://www.python.org/dev/peps/pep-0008/). It also checks for potential problems such as imports of deprecated modules. PEP8 is a widely known Python style guide, recommended and written by the developer of Python Guido van Rossum himself. `SoftWipe` received a Pylint score of 7.17/10. However, this is mainly due to recommendations that, based on our personal assessment, would reduce the readability of our code. The complete output of Pylint is part of the Appendix 6.3.

5.2 Pyflakes

Pyflakes is a static analyzer that focuses on finding source code errors. It does not assess coding style. This analyzer did not detect any issues with our `SoftWipe` code.

5.3 Radon

Radon computes multiple code metrics that quantify code quality. These metrics are: Cyclomatic Complexity (CC), Halstead Metrics, and the Maintainability Index. We explain the CC in Section 1.4. Radon computes an average CC of 3.77, which yields the score "A" (low risk, simple blocks). The Halstead Metrics assume that programs are built out of operators and operands and uses these to estimate the difficulty to understand the program, the effort and time needed for the implementation, and the number of bugs in the implementation. We provide the calculated values in Table 5. The Maintainability Index estimates the difficulty to maintain (i.e., support, change, adapt) the program. For its calculation it considers CC, lines of code, the Halstead Volume (part of Halstead Metrics) as well as the percentage of comment lines. We receive an "A" (very high maintainability) for all SoftWipe files.

**file name**	**difficulty**	**effort**	**time program**	**bugs**
analysis_tools.py	8.0	22451	1247	0.93
automatic_tool_installation.py	4.23	1605	89	0.12
calculate_score_table.py	7.85	9688	538	0.41
classifications.py	0	0	0	0
compare_results.py	9.9	9833	546	0.33
compile_phase.py	8.4	6810	378	0.27
execution_phase.py	3.55	664	37	0.06
output_classes.py	3.01	3323	184	0.37
recalculate_scores_from_table.py	9.7	9596	533	0.33
scoring.py	9.5	15190	843	0.53
softwipe.py	5.25	3018	167	0.19
strings.py	0.67	540	30	0.27
tools_info.py	0	0	0	0
util.py	4.22	1654	91	0.13

Table 5: Radon Halstead Metrics for SoftWipe

5.4 Lizard

We also used Lizard (for a description see Section 1.4) to assess the code quality of SoftWipe, since Lizard is not limited to C/C++. Based on Lizard we computed absolute scores (see Section 2.2) for the cyclomatic complexity, the Lizard warnings, and the unique code. The scores are 9/10, 8.5/10, and 9.7/10 respectively. The complete output is part of the Appendix 6.4.

References

[1] Thomas J. McCabe. A complexity measure. *IEEE Transactions on Software Engineering*, SE-2:308–320, 1976.

[2] John W Tukey. *Exploratory Data Analysis*. Addison-Wesley, 1997. ISBN 978-0-201-07616-5.

6 Appendix

6.1 Benchmark Scores

program	overall	relative score	compiler and sanitizer	assertions	cppcheck	clang tidy	cyclomatic complexity	lizard warnings	unique	kwstyle	infer	test count
genesis-0.24.0	9.0	9.0	9.9	8.7	8.3	9.2	9.0	9.4	8.2	8.2	N/A	10.0
fastspar	8.2	8.4	9.2	2.0	9.9	9.9	8.8	7.9	8.8	6.4	9.2	10.0
parallel-STL	7.4	7.0	10.0	0.4	7.1	5.6	9.3	9.9	6.3	8.4	N/A	10.0
raxml-ng_v1.0.1	7.3	7.5	9.7	4.2	5.3	9.0	7.9	6.6	4.0	9.2	N/A	10.0
kahypar	7.3	7.8	6.7	2.4	8.0	N/A	9.2	9.6	3.3	9.1	N/A	10.0
ExpansionHunter-4.0.2	7.2	7.2	8.7	1.8	8.6	9.4	8.9	9.1	0.4	7.9	N/A	10.0
naf-1.1.0/unnaf	6.8	7.0	9.9	4.0	9.9	10.0	6.9	7.5	7.2	3.3	9.5	0.0
bindash-1.0	6.8	6.7	6.8	8.8	3.4	7.1	8.7	9.5	8.2	8.5	N/A	0.0
dawg-1.2	6.8	6.9	10.0	0.0	7.4	10.0	8.4	8.1	7.9	9.1	N/A	0.0
naf-1.1.0/ennaf	6.8	7.0	9.8	10.0	9.6	10.0	7.2	6.7	0.0	5.2	9.0	0.0
virulign-1.0.1	6.5	6.6	7.9	3.4	8.6	9.0	7.3	5.8	7.5	9.3	N/A	0.0
Treerecs-v1.2	6.5	6.8	5.0	1.8	6.0	8.6	9.0	9.0	1.6	7.5	N/A	10.0
glucose-3-drup	6.4	6.9	7.8	10.0	3.8	9.4	8.7	8.4	8.5	1.4	N/A	0.0
swarm-3.0.0	6.3	6.1	10.0	0.3	9.2	3.8	8.0	7.7	4.3	9.9	10.0	0.0
RepeatsCounter	6.3	6.2	4.2	0.0	7.8	6.8	9.0	10.0	9.3	9.5	N/A	0.0
samtools-1.11	6.0	6.5	7.4	1.2	7.0	9.1	3.8	2.2	8.2	6.3	4.3	9.9
bpp-4.3.8	5.9	6.3	9.5	9.3	5.8	8.9	2.8	2.0	6.6	9.3	4.9	0.0
ntEdit-1.2.3	5.7	6.1	6.9	0.0	6.9	9.7	7.9	6.7	3.8	7.7	8.1	0.0
prank-msa	5.5	5.9	2.2	5.1	9.8	9.0	7.0	6.6	1.4	5.8	8.0	0.0
IQ-TREE-2.0.6	5.5	5.6	0.2	2.5	3.2	7.8	8.2	7.7	5.3	6.6	N/A	7.7
dna-nn-0.1	5.3	5.1	5.2	4.1	6.7	6.0	6.7	5.0	6.1	7.8	N/A	0.0
openmp	5.2	5.4	2.9	0.9	0.1	1.5	8.1	7.3	7.6	8.3	N/A	10.0
ngsTools/ngsLD	5.2	4.9	8.2	0.0	7.1	6.1	5.0	3.9	8.3	7.9	N/A	0.0
emeraLD	5.2	5.3	0.1	0.0	8.7	8.4	6.3	5.3	9.0	8.6	N/A	0.0
defor	5.1	5.3	0.0	0.0	4.7	9.4	6.9	6.4	9.0	9.4	N/A	0.0
copmem-0.2	5.1	5.2	10.0	0.2	7.0	8.6	8.5	7.8	4.2	4.5	0.0	0.0
BGSA-1.0	5.0	5.4	4.3	0.0	0.2	10.0	7.5	6.8	8.2	9.4	3.8	0.0
phyml-3.3.20200621	4.9	5.3	9.0	5.5	4.4	8.1	4.3	2.7	5.9	3.7	5.0	0.0
dr_sasa_n	4.8	5.5	0.4	0.0	9.8	10.0	2.3	1.6	9.2	9.9	N/A	0.0
HLA-LA	4.8	5.6	5.5	10.0	3.0	9.5	5.0	4.1	2.9	3.1	4.9	0.0
SF2	4.6	4.9	10.0	1.3	2.4	7.9	3.0	0.8	3.3	6.9	10.0	0.0
Seq-Gen-1.3.4	4.6	4.9	7.8	0.0	6.5	8.3	5.7	5.2	8.9	2.5	0.7	0.0
clustal-omega-1.2.4	4.4	5.0	5.5	3.1	6.5	8.8	3.9	2.5	5.3	3.9	N/A	0.2
Gadget-2.0.7	4.4	4.6	10.0	0.0	1.5	10.0	0.4	0.1	5.4	9.1	N/A	3.0
cellcoal-1.0.0	4.4	4.2	9.3	0.0	5.7	7.5	0.8	0.1	7.2	6.9	6.3	0.0
minimap2-2.17	4.3	4.6	2.7	2.6	4.7	6.6	6.1	5.2	8.0	5.1	2.6	0.0
ms	4.3	4.6	6.6	0.0	0.0	10.0	6.2	5.3	6.4	0.0	8.9	0.0
MrBayes-3.2.7a	3.9	3.9	9.3	1.4	8.1	7.1	0.0	0.1	3.8	4.5	5.2	0.0
prequal	3.9	4.3	0.0	5.9	0.2	9.9	6.0	4.0	1.0	2.8	8.8	0.0
cryfa-18.06	3.8	4.1	5.5	2.0	0.0	9.7	5.9	5.5	6.0	0.0	N/A	0.0
vsearch-2.15.1	3.8	4.4	4.0	0.0	7.0	1.1	5.0	3.9	5.6	9.7	1.4	0.0
sumo	3.7	3.9	0.0	1.2	6.1	9.4	8.0	7.4	0.0	0.5	N/A	0.7

PopLDdecay	3.6	3.7	7.9	0.0	8.9	10.0	0.1	0.0	0.0	0.0	8.6	0.0
crisflash	3.5	4.3	1.0	0.0	3.3	10.0	5.4	4.1	6.2	4.9	0.0	0.0
athena-public-version-21.0	3.4	3.7	0.8	0.0	0.1	8.2	4.5	2.5	0.6	9.1	8.2	0.3
mafft-7.475	3.1	3.1	8.2	0.0	6.2	7.8	0.3	0.4	0.7	6.5	0.3	0.8
covid-sim-0.13.0	2.7	3.0	6.7	0.0	5.2	0.0	0.0	0.0	7.3	0.3	N/A	4.9
INDELibleV1.03	1.9	2.3	0.8	0.0	0.5	9.3	0.7	0.8	6.7	0.0	0.0	0.0

6.2 Benchmark Absolute Values

program	loc	functions	compiler	sanitizer	assertions	cppcheck	clang tidy	cyclomatic complexity	lizard warnings	unique	kwstyle	infer	test count
sumo	514811	23788	2016523	0	995	10957	9057	4.6	1285	0.7079	43493	N/A	2563
IQ-TREE-2.0.6	220709	10930	168082	0	852	8001	13767	4.2	527	0.9098	5827	N/A	10994
Treerecs-v1.2	171121	10189	48109	0	483	3790	6861	2.4	210	0.845	3314	N/A	64810
dr_sasa_n	146963	86	97408	0	0	185	22	11.5	15	0.9968	94	N/A	0
kahypar	109786	9732	20475	0	417	1207	N/A	1.7	72	0.8796	751	N/A	72051
MrBayes-3.2.7a	95597	962	3941	4	205	1017	7969	22.6	287	0.8872	3984	598	0
openmp	91040	3530	35423	0	127	6812	22105	4.4	196	0.9479	1157	N/A	16895
raxml-ng_v1.0.1	87135	2545	1302	0	572	2282	2602	4.7	181	0.8903	531	N/A	13049
samtools-1.11	78959	2321	11592	0	151	1317	2038	9.5	364	0.9626	2264	572	7626
mafft-7.475	77251	932	7790	0	0	1641	4849	17.8	226	0.81	2098	1228	405
ExpansionHunter-4.0.2	72944	3945	5275	5	208	584	1296	2.8	74	0.7912	1157	N/A	18758
phyml-3.3.20200621	70845	1609	4030	1	596	2161	3762	9.0	235	0.9188	3301	464	0
athena-public-version-21.0	65302	1509	37159	1	3	5093	3325	8.8	229	0.8005	463	157	131
genesis-0.24.0	62886	3855	434	0	859	576	1472	2.4	49	0.9608	885	N/A	7658
bpp-4.3.8	41109	793	1180	0	646	947	1314	10.8	129	0.9305	210	272	0
clustal-omega-1.2.4	34160	883	8764	0	162	669	1133	9.4	133	0.9106	1557	N/A	42
vsearch-2.15.1	24384	506	8086	0	0	409	6409	8.2	62	0.9142	65	277	0
prank-msa	24023	756	10245	0	188	33	660	6.0	54	0.8378	773	63	0
HLA-LA	23811	462	6133	0	1653	884	337	8.3	55	0.872	1217	158	0
covid-sim-0.13.0	13200	124	2451	0	0	353	9280	32.5	42	0.9434	1255	N/A	433
Gadget-2.0.7	12589	148	0	0	0	594	4	16.9	47	0.9117	83	N/A	257
fastspar	11346	90	525	0	35	9	23	3.1	4	0.9779	310	12	9933
cellcoal-1.0.0	11000	66	420	0	0	264	793	14.7	21	0.9406	264	53	0
parallel-STL	10380	1162	0	0	7	168	1303	1.6	2	0.9247	128	N/A	6617
INDELibleV1.03	9697	216	5480	0	0	596	199	14.9	45	0.9321	4252	303	0
minimap2-2.17	8841	339	3522	0	35	257	859	7.1	34	0.9569	334	82	0
swarm-3.0.0	7092	212	0	0	3	33	1204	4.6	10	0.8945	7	0	0
dawg-1.2	7058	256	0	0	0	103	0	3.9	10	0.9539	47	N/A	0
PopLDdecay	6557	57	786	0	0	39	3	19.5	20	0.4369	1418	12	0
SF2	5337	121	0	0	11	217	312	10.5	25	0.8789	129	0	0
glucose-3-drup	4772	479	603	0	149	159	78	3.3	16	0.9705	318	N/A	0
dna-nn-0.1	4768	210	1295	1	30	88	541	6.4	22	0.923	82	N/A	0
ngsTools/ngsLD	4373	113	434	0	0	70	487	8.3	14	0.9643	69	N/A	0
Seq-Gen-1.3.4	3980	120	490	0	0	77	195	7.5	12	0.9828	222	53	0
crisflash	3279	84	1743	0	0	116	0	7.8	10	0.9238	128	135	0
copmem-0.2	3026	133	4	0	1	51	123	3.7	6	0.8939	125	84	0
prequal	2600	99	2410	0	23	182	4	7.2	12	0.8228	139	4	0
ntEdit-1.2.3	2365	87	418	0	0	41	23	4.8	6	0.8867	42	6	0
cryfa-18.06	2216	74	560	5	7	403	20	7.3	7	0.9213	372	N/A	0
ms	2182	71	423	1	0	278	0	7.0	7	0.9263	641	3	0
emeraLD	1642	51	1338	0	0	12	74	6.8	5	0.988	18	N/A	0
bindash-1.0	1622	88	293	0	23	57	133	3.2	1	0.963	19	N/A	0
naf-1.1.0/unnaf	1620	77	7	2	10	1	0	6.1	4	0.9415	80	1	0
naf-1.1.0/ennaf	1615	73	13	1	78	4	0	5.7	5	0.6041	60	2	0
BGSA-1.0	1405	30	445	0	0	103	0	5.3	2	0.9621	7	11	0
virulign-1.0.1	1149	46	139	0	6	9	33	5.6	4	0.9464	6	N/A	0

defor	695	27	1228	0	0	20	11	6.2	2	0.9876	3	N/A	0
RepeatsCounter	243	19	76	2	0	3	22	2.4	0	1.0	1	N/A	0

6.3 Pylint Output

The output of Pylint is also available at
https://github.com/adrianzap/softwipe/blob/master/softwipe_code_quality/pylint_results.txt.

```
    {************* Module analysis_tools
analysis_tools.py:33:0: C0301: Line too long (114/100) (line-too-long)
analysis_tools.py:65:0: C0301: Line too long (117/100) (line-too-long)
analysis_tools.py:74:0: C0301: Line too long (106/100) (line-too-long)
analysis_tools.py:78:0: C0301: Line too long (106/100) (line-too-long)
analysis_tools.py:81:0: C0301: Line too long (115/100) (line-too-long)
analysis_tools.py:85:0: C0301: Line too long (107/100) (line-too-long)
analysis_tools.py:89:0: C0301: Line too long (107/100) (line-too-long)
analysis_tools.py:103:0: C0301: Line too long (112/100) (line-too-long)
analysis_tools.py:107:0: C0301: Line too long (117/100) (line-too-long)
analysis_tools.py:109:0: C0301: Line too long (111/100) (line-too-long)
analysis_tools.py:110:0: C0301: Line too long (115/100) (line-too-long)
analysis_tools.py:111:0: C0301: Line too long (112/100) (line-too-long)
analysis_tools.py:140:0: C0301: Line too long (118/100) (line-too-long)
analysis_tools.py:142:0: C0301: Line too long (112/100) (line-too-long)
analysis_tools.py:144:0: C0301: Line too long (102/100) (line-too-long)
analysis_tools.py:149:0: C0301: Line too long (103/100) (line-too-long)
analysis_tools.py:150:0: C0301: Line too long (109/100) (line-too-long)
analysis_tools.py:187:0: C0301: Line too long (117/100) (line-too-long)
analysis_tools.py:210:0: C0301: Line too long (114/100) (line-too-long)
analysis_tools.py:212:0: C0301: Line too long (106/100) (line-too-long)
analysis_tools.py:217:0: C0301: Line too long (113/100) (line-too-long)
analysis_tools.py:234:0: C0301: Line too long (106/100) (line-too-long)
analysis_tools.py:248:0: C0301: Line too long (112/100) (line-too-long)
analysis_tools.py:255:0: C0301: Line too long (120/100) (line-too-long)
analysis_tools.py:257:0: C0301: Line too long (116/100) (line-too-long)
analysis_tools.py:274:0: C0301: Line too long (107/100) (line-too-long)
analysis_tools.py:276:0: C0301: Line too long (104/100) (line-too-long)
analysis_tools.py:309:0: C0301: Line too long (108/100) (line-too-long)
analysis_tools.py:326:0: C0301: Line too long (102/100) (line-too-long)
analysis_tools.py:337:0: C0301: Line too long (107/100) (line-too-long)
analysis_tools.py:338:0: C0301: Line too long (117/100) (line-too-long)
analysis_tools.py:339:0: C0301: Line too long (115/100) (line-too-long)
analysis_tools.py:346:0: C0301: Line too long (108/100) (line-too-long)
analysis_tools.py:347:0: C0301: Line too long (114/100) (line-too-long)
analysis_tools.py:379:0: C0301: Line too long (110/100) (line-too-long)
analysis_tools.py:394:0: C0301: Line too long (108/100) (line-too-long)
analysis_tools.py:397:0: C0301: Line too long (105/100) (line-too-long)
analysis_tools.py:405:0: C0301: Line too long (117/100) (line-too-long)
analysis_tools.py:426:0: C0301: Line too long (102/100) (line-too-long)
analysis_tools.py:463:0: C0301: Line too long (118/100) (line-too-long)
analysis_tools.py:469:0: C0301: Line too long (118/100) (line-too-long)
analysis_tools.py:487:0: C0301: Line too long (108/100) (line-too-long)
analysis_tools.py:488:0: C0301: Line too long (101/100) (line-too-long)
analysis_tools.py:538:0: C0301: Line too long (109/100) (line-too-long)
analysis_tools.py:565:0: C0301: Line too long (103/100) (line-too-long)
analysis_tools.py:575:0: C0301: Line too long (105/100) (line-too-long)
analysis_tools.py:576:0: C0301: Line too long (111/100) (line-too-long)
analysis_tools.py:600:0: C0301: Line too long (112/100) (line-too-long)
analysis_tools.py:604:0: C0301: Line too long (119/100) (line-too-long)
analysis_tools.py:607:0: C0301: Line too long (105/100) (line-too-long)
analysis_tools.py:608:0: C0301: Line too long (111/100) (line-too-long)
analysis_tools.py:665:0: C0301: Line too long (105/100) (line-too-long)
analysis_tools.py:701:0: C0301: Line too long (120/100) (line-too-long)
analysis_tools.py:750:0: C0301: Line too long (120/100) (line-too-long)
analysis_tools.py:776:0: C0301: Line too long (118/100) (line-too-long)
analysis_tools.py:53:2: W0511: TODO: put compilation into classes (?) (fixme)
analysis_tools.py:393:2: W0511: TODO: find out the purpose of the --template=cppcheck1' which broke the output (fixme)
analysis_tools.py:402:2: W0511: TODO: check this again (fixme)
analysis_tools.py:632:2: W0511: TODO: maybe fix the error handling differently (not by the --keep-going flag) (fixme)
analysis_tools.py:664:2: W0511: TODO: make and print filename to user (fixme)
analysis_tools.py:749:2: W0511: TODO: make and print filename to user (fixme)
```

```
analysis_tools.py:23:0: C0115: Missing class docstring (missing-class-docstring)
analysis_tools.py:28:12: W0613: Unused argument 'data' (unused-argument)
analysis_tools.py:28:18: W0613: Unused argument 'skip_on_failure' (unused-argument)
analysis_tools.py:38:4: C0116: Missing function or method docstring (missing-function-docstring)
analysis_tools.py:42:0: C0115: Missing class docstring (missing-class-docstring)
analysis_tools.py:52:0: C0115: Missing class docstring (missing-class-docstring)
analysis_tools.py:55:4: W0221: Parameters differ from overridden 'run' method (arguments-differ)
analysis_tools.py:69:-1: W0105: String statement has no effect (pointless-string-statement)
analysis_tools.py:82:72: E0602: Undefined variable 'cpp' (undefined-variable)
analysis_tools.py:99:0: C0115: Missing class docstring (missing-class-docstring)
analysis_tools.py:160:0: C0115: Missing class docstring (missing-class-docstring)
analysis_tools.py:223:4: W0221: Parameters differ from overridden 'run' method (arguments-differ)
analysis_tools.py:223:4: R0914: Too many local variables (17/15) (too-many-locals)
analysis_tools.py:238:-1: W0105: String statement has no effect (pointless-string-statement)
analysis_tools.py:259:15: W0703: Catching too general exception Exception (broad-except)
analysis_tools.py:286:0: C0115: Missing class docstring (missing-class-docstring)
analysis_tools.py:336:-1: W0105: String statement has no effect (pointless-string-statement)
analysis_tools.py:351:15: W0703: Catching too general exception Exception (broad-except)
analysis_tools.py:372:0: C0115: Missing class docstring (missing-class-docstring)
analysis_tools.py:374:4: C0116: Missing function or method docstring (missing-function-docstring)
analysis_tools.py:387:4: R0914: Too many local variables (19/15) (too-many-locals)
analysis_tools.py:414:15: W0703: Catching too general exception Exception (broad-except)
analysis_tools.py:436:0: C0115: Missing class docstring (missing-class-docstring)
analysis_tools.py:474:19: W0703: Catching too general exception Exception (broad-except)
analysis_tools.py:498:0: C0115: Missing class docstring (missing-class-docstring)
analysis_tools.py:573:8: C0103: Variable name "e" doesn't conform to snake_case naming style (invalid-name)
analysis_tools.py:605:8: C0103: Variable name "e" doesn't conform to snake_case naming style (invalid-name)
analysis_tools.py:615:4: R0914: Too many local variables (19/15) (too-many-locals)
analysis_tools.py:646:15: W0703: Catching too general exception Exception (broad-except)
analysis_tools.py:676:0: C0115: Missing class docstring (missing-class-docstring)
analysis_tools.py:678:4: C0116: Missing function or method docstring (missing-function-docstring)
analysis_tools.py:681:16: C0103: Variable name "w" doesn't conform to snake_case naming style (invalid-name)
analysis_tools.py:687:4: C0116: Missing function or method docstring (missing-function-docstring)
analysis_tools.py:700:4: R0914: Too many local variables (17/15) (too-many-locals)
analysis_tools.py:729:8: C0103: Variable name "e1" doesn't conform to snake_case naming style (invalid-name)
analysis_tools.py:762:0: C0115: Missing class docstring (missing-class-docstring)
************* Module automatic_tool_installation
automatic_tool_installation.py:45:0: C0301: Line too long (109/100) (line-too-long)
automatic_tool_installation.py:59:0: C0301: Line too long (104/100) (line-too-long)
automatic_tool_installation.py:95:0: C0301: Line too long (104/100) (line-too-long)
automatic_tool_installation.py:96:0: C0301: Line too long (109/100) (line-too-long)
automatic_tool_installation.py:117:0: C0301: Line too long (112/100) (line-too-long)
automatic_tool_installation.py:125:0: C0301: Line too long (112/100) (line-too-long)
automatic_tool_installation.py:138:0: C0301: Line too long (110/100) (line-too-long)
automatic_tool_installation.py:183:0: C0301: Line too long (108/100) (line-too-long)
automatic_tool_installation.py:115:2: W0511: TODO: properly select newest version (fixme)
automatic_tool_installation.py:25:8: C0415: Import outside toplevel (distro) (import-outside-toplevel)
automatic_tool_installation.py:33:0: C0116: Missing function or method docstring (missing-function-docstring)
automatic_tool_installation.py:42:0: C0116: Missing function or method docstring (missing-function-docstring)
automatic_tool_installation.py:50:0: C0116: Missing function or method docstring (missing-function-docstring)
automatic_tool_installation.py:54:4: W1510: Using subprocess.run without explicitly set `check` is not recommended.
(subprocess-run-check)
automatic_tool_installation.py:58:0: C0116: Missing function or method docstring (missing-function-docstring)
automatic_tool_installation.py:62:8: R1705: Unnecessary "elif" after "return" (no-else-return)
automatic_tool_installation.py:64:12: W1510: Using subprocess.run without explicitly set `check` is not recommended.
(subprocess-run-check)
automatic_tool_installation.py:76:0: C0116: Missing function or method docstring (missing-function-docstring)
automatic_tool_installation.py:85:0: C0116: Missing function or method docstring (missing-function-docstring)
automatic_tool_installation.py:91:4: W1510: Using subprocess.run without explicitly set `check` is not recommended.
(subprocess-run-check)
automatic_tool_installation.py:101:0: C0116: Missing function or method docstring (missing-function-docstring)
automatic_tool_installation.py:112:0: C0116: Missing function or method docstring (missing-function-docstring)
automatic_tool_installation.py:148:0: C0116: Missing function or method docstring (missing-function-docstring)
automatic_tool_installation.py:168:0: C0116: Missing function or method docstring (missing-function-docstring)
************* Module calculate_score_table
calculate_score_table.py:4:0: C0301: Line too long (117/100) (line-too-long)
calculate_score_table.py:5:0: C0301: Line too long (116/100) (line-too-long)
calculate_score_table.py:18:0: C0301: Line too long (116/100) (line-too-long)
```

```
calculate_score_table.py:19:0: C0301: Line too long (114/100) (line-too-long)
calculate_score_table.py:21:0: C0301: Line too long (120/100) (line-too-long)
calculate_score_table.py:22:0: C0301: Line too long (113/100) (line-too-long)
calculate_score_table.py:23:0: C0301: Line too long (119/100) (line-too-long)
calculate_score_table.py:26:0: C0301: Line too long (104/100) (line-too-long)
calculate_score_table.py:30:0: C0301: Line too long (111/100) (line-too-long)
calculate_score_table.py:51:0: C0301: Line too long (110/100) (line-too-long)
calculate_score_table.py:57:0: C0301: Line too long (118/100) (line-too-long)
calculate_score_table.py:59:0: C0301: Line too long (117/100) (line-too-long)
calculate_score_table.py:60:0: C0301: Line too long (115/100) (line-too-long)
calculate_score_table.py:61:0: C0301: Line too long (118/100) (line-too-long)
calculate_score_table.py:64:0: C0301: Line too long (115/100) (line-too-long)
calculate_score_table.py:81:0: C0301: Line too long (116/100) (line-too-long)
calculate_score_table.py:82:0: C0301: Line too long (118/100) (line-too-long)
calculate_score_table.py:85:0: C0301: Line too long (126/100) (line-too-long)
calculate_score_table.py:87:0: C0301: Line too long (117/100) (line-too-long)
calculate_score_table.py:88:0: C0301: Line too long (108/100) (line-too-long)
calculate_score_table.py:99:0: C0301: Line too long (106/100) (line-too-long)
calculate_score_table.py:100:0: C0301: Line too long (105/100) (line-too-long)
calculate_score_table.py:108:0: C0301: Line too long (106/100) (line-too-long)
calculate_score_table.py:117:0: C0301: Line too long (104/100) (line-too-long)
calculate_score_table.py:134:0: C0301: Line too long (107/100) (line-too-long)
calculate_score_table.py:151:0: C0301: Line too long (117/100) (line-too-long)
calculate_score_table.py:152:0: C0301: Line too long (108/100) (line-too-long)
calculate_score_table.py:164:0: C0301: Line too long (106/100) (line-too-long)
calculate_score_table.py:165:0: C0301: Line too long (105/100) (line-too-long)
calculate_score_table.py:168:0: C0301: Line too long (106/100) (line-too-long)
calculate_score_table.py:180:0: C0301: Line too long (104/100) (line-too-long)
calculate_score_table.py:198:0: C0301: Line too long (119/100) (line-too-long)
calculate_score_table.py:199:0: C0301: Line too long (103/100) (line-too-long)
calculate_score_table.py:246:0: C0301: Line too long (120/100) (line-too-long)
calculate_score_table.py:247:0: C0301: Line too long (125/100) (line-too-long)
calculate_score_table.py:266:0: C0301: Line too long (108/100) (line-too-long)
calculate_score_table.py:267:0: C0301: Line too long (117/100) (line-too-long)
calculate_score_table.py:268:0: C0301: Line too long (107/100) (line-too-long)
calculate_score_table.py:273:0: C0301: Line too long (104/100) (line-too-long)
calculate_score_table.py:275:0: C0301: Line too long (105/100) (line-too-long)
calculate_score_table.py:277:0: C0301: Line too long (101/100) (line-too-long)
calculate_score_table.py:279:0: C0301: Line too long (107/100) (line-too-long)
calculate_score_table.py:281:0: C0301: Line too long (117/100) (line-too-long)
calculate_score_table.py:283:0: C0301: Line too long (112/100) (line-too-long)
calculate_score_table.py:291:0: C0301: Line too long (107/100) (line-too-long)
calculate_score_table.py:333:0: C0301: Line too long (112/100) (line-too-long)
calculate_score_table.py:354:0: C0301: Line too long (103/100) (line-too-long)
calculate_score_table.py:24:2: W0511: TODO: add SPRING (fixme)
calculate_score_table.py:56:0: C0116: Missing function or method docstring (missing-function-docstring)
calculate_score_table.py:75:0: C0116: Missing function or method docstring (missing-function-docstring)
calculate_score_table.py:75:0: R0914: Too many local variables (18/15) (too-many-locals)
calculate_score_table.py:98:45: C0321: More than one statement on a single line (multiple-statements)
calculate_score_table.py:99:59: C0321: More than one statement on a single line (multiple-statements)
calculate_score_table.py:102:46: C0321: More than one statement on a single line (multiple-statements)
calculate_score_table.py:107:47: C0321: More than one statement on a single line (multiple-statements)
calculate_score_table.py:108:59: C0321: More than one statement on a single line (multiple-statements)
calculate_score_table.py:111:45: C0321: More than one statement on a single line (multiple-statements)
calculate_score_table.py:114:47: C0321: More than one statement on a single line (multiple-statements)
calculate_score_table.py:117:58: C0321: More than one statement on a single line (multiple-statements)
calculate_score_table.py:120:52: C0321: More than one statement on a single line (multiple-statements)
calculate_score_table.py:123:43: C0321: More than one statement on a single line (multiple-statements)
calculate_score_table.py:126:44: C0321: More than one statement on a single line (multiple-statements)
calculate_score_table.py:129:42: C0321: More than one statement on a single line (multiple-statements)
calculate_score_table.py:132:46: C0321: More than one statement on a single line (multiple-statements)
calculate_score_table.py:75:0: R0912: Too many branches (25/12) (too-many-branches)
calculate_score_table.py:75:0: R0915: Too many statements (56/50) (too-many-statements)
calculate_score_table.py:138:0: C0116: Missing function or method docstring (missing-function-docstring)
calculate_score_table.py:138:0: R0914: Too many local variables (22/15) (too-many-locals)
calculate_score_table.py:163:45: C0321: More than one statement on a single line (multiple-statements)
calculate_score_table.py:164:59: C0321: More than one statement on a single line (multiple-statements)
calculate_score_table.py:167:46: C0321: More than one statement on a single line (multiple-statements)
```

```
calculate_score_table.py:168:59: C0321: More than one statement on a single line (multiple-statements)
calculate_score_table.py:171:47: C0321: More than one statement on a single line (multiple-statements)
calculate_score_table.py:174:45: C0321: More than one statement on a single line (multiple-statements)
calculate_score_table.py:177:47: C0321: More than one statement on a single line (multiple-statements)
calculate_score_table.py:180:58: C0321: More than one statement on a single line (multiple-statements)
calculate_score_table.py:184:52: C0321: More than one statement on a single line (multiple-statements)
calculate_score_table.py:187:43: C0321: More than one statement on a single line (multiple-statements)
calculate_score_table.py:190:44: C0321: More than one statement on a single line (multiple-statements)
calculate_score_table.py:193:42: C0321: More than one statement on a single line (multiple-statements)
calculate_score_table.py:196:46: C0321: More than one statement on a single line (multiple-statements)
calculate_score_table.py:138:0: R0912: Too many branches (26/12) (too-many-branches)
calculate_score_table.py:138:0: R0915: Too many statements (59/50) (too-many-statements)
calculate_score_table.py:202:0: C0116: Missing function or method docstring (missing-function-docstring)
calculate_score_table.py:202:0: R0914: Too many local variables (30/15) (too-many-locals)
calculate_score_table.py:294:65: C0201: Consider iterating the dictionary directly instead of calling .keys()
(consider-iterating-dictionary)
calculate_score_table.py:296:39: C0321: More than one statement on a single line (multiple-statements)
calculate_score_table.py:202:0: R0912: Too many branches (20/12) (too-many-branches)
calculate_score_table.py:202:0: R0915: Too many statements (52/50) (too-many-statements)
calculate_score_table.py:308:0: C0116: Missing function or method docstring (missing-function-docstring)
calculate_score_table.py:308:0: R0912: Too many branches (15/12) (too-many-branches)
calculate_score_table.py:348:0: C0116: Missing function or method docstring (missing-function-docstring)
calculate_score_table.py:351:15: R1719: The if expression can be replaced with 'bool(test)' (simplifiable-if-expression)
calculate_score_table.py:352:25: R1719: The if expression can be replaced with 'bool(test)' (simplifiable-if-expression)
************* Module classifications
classifications.py:140:0: C0301: Line too long (118/100) (line-too-long)
classifications.py:141:0: C0301: Line too long (103/100) (line-too-long)
************* Module compare_results
compare_results.py:4:0: C0301: Line too long (115/100) (line-too-long)
compare_results.py:17:0: C0301: Line too long (107/100) (line-too-long)
compare_results.py:19:0: C0301: Line too long (117/100) (line-too-long)
compare_results.py:20:0: C0301: Line too long (110/100) (line-too-long)
compare_results.py:21:0: C0301: Line too long (114/100) (line-too-long)
compare_results.py:22:0: C0301: Line too long (117/100) (line-too-long)
compare_results.py:23:0: C0301: Line too long (103/100) (line-too-long)
compare_results.py:25:0: C0301: Line too long (109/100) (line-too-long)
compare_results.py:27:0: C0301: Line too long (115/100) (line-too-long)
compare_results.py:29:0: C0301: Line too long (117/100) (line-too-long)
compare_results.py:31:0: C0301: Line too long (115/100) (line-too-long)
compare_results.py:32:0: C0301: Line too long (116/100) (line-too-long)
compare_results.py:33:0: C0301: Line too long (119/100) (line-too-long)
compare_results.py:34:0: C0301: Line too long (114/100) (line-too-long)
compare_results.py:37:0: C0301: Line too long (118/100) (line-too-long)
compare_results.py:38:0: C0301: Line too long (116/100) (line-too-long)
compare_results.py:60:0: C0301: Line too long (117/100) (line-too-long)
compare_results.py:61:0: C0301: Line too long (113/100) (line-too-long)
compare_results.py:83:0: C0301: Line too long (111/100) (line-too-long)
compare_results.py:155:0: C0301: Line too long (110/100) (line-too-long)
compare_results.py:160:0: C0301: Line too long (111/100) (line-too-long)
compare_results.py:16:0: C0116: Missing function or method docstring (missing-function-docstring)
compare_results.py:44:0: C0116: Missing function or method docstring (missing-function-docstring)
compare_results.py:60:8: W0632: Possible unbalanced tuple unpacking with sequence defined at line 134
of calculate_score_table: left side has 10 label(s), right side has 11 value(s) (unbalanced-tuple-unpacking)
compare_results.py:61:34: W0612: Unused variable 'failed_tools' (unused-variable)
compare_results.py:83:18: R1719: The if expression can be replaced with 'test' (simplifiable-if-expression)
compare_results.py:90:0: C0116: Missing function or method docstring (missing-function-docstring)
compare_results.py:90:21: W0622: Redefining built-in 'list' (redefined-builtin)
compare_results.py:101:0: C0116: Missing function or method docstring (missing-function-docstring)
compare_results.py:114:0: C0116: Missing function or method docstring (missing-function-docstring)
compare_results.py:114:23: W0622: Redefining built-in 'list' (redefined-builtin)
compare_results.py:119:4: C0103: Variable name "q1" doesn't conform to snake_case naming style (invalid-name)
compare_results.py:120:4: C0103: Variable name "q3" doesn't conform to snake_case naming style (invalid-name)
compare_results.py:131:0: C0116: Missing function or method docstring (missing-function-docstring)
compare_results.py:149:0: C0116: Missing function or method docstring (missing-function-docstring)
compare_results.py:200:0: C0116: Missing function or method docstring (missing-function-docstring)
compare_results.py:213:0: C0116: Missing function or method docstring (missing-function-docstring)
compare_results.py:217:0: C0116: Missing function or method docstring (missing-function-docstring)
************* Module compile_phase
```

```
compile_phase.py:35:0: C0301: Line too long (117/100) (line-too-long)
compile_phase.py:39:0: C0301: Line too long (118/100) (line-too-long)
compile_phase.py:40:0: C0301: Line too long (112/100) (line-too-long)
compile_phase.py:41:0: C0301: Line too long (105/100) (line-too-long)
compile_phase.py:43:0: C0301: Line too long (115/100) (line-too-long)
compile_phase.py:45:0: C0301: Line too long (114/100) (line-too-long)
compile_phase.py:46:0: C0301: Line too long (114/100) (line-too-long)
compile_phase.py:47:0: C0301: Line too long (116/100) (line-too-long)
compile_phase.py:51:0: C0301: Line too long (118/100) (line-too-long)
compile_phase.py:61:0: C0301: Line too long (117/100) (line-too-long)
compile_phase.py:66:0: C0301: Line too long (110/100) (line-too-long)
compile_phase.py:73:0: C0301: Line too long (117/100) (line-too-long)
compile_phase.py:74:0: C0301: Line too long (114/100) (line-too-long)
compile_phase.py:81:0: C0301: Line too long (116/100) (line-too-long)
compile_phase.py:83:0: C0301: Line too long (118/100) (line-too-long)
compile_phase.py:88:0: C0301: Line too long (116/100) (line-too-long)
compile_phase.py:98:0: C0301: Line too long (112/100) (line-too-long)
compile_phase.py:141:0: C0301: Line too long (111/100) (line-too-long)
compile_phase.py:151:0: C0301: Line too long (107/100) (line-too-long)
compile_phase.py:153:0: C0301: Line too long (111/100) (line-too-long)
compile_phase.py:155:0: C0301: Line too long (109/100) (line-too-long)
compile_phase.py:170:0: C0301: Line too long (110/100) (line-too-long)
compile_phase.py:173:0: C0301: Line too long (106/100) (line-too-long)
compile_phase.py:184:0: C0301: Line too long (104/100) (line-too-long)
compile_phase.py:185:0: C0301: Line too long (113/100) (line-too-long)
compile_phase.py:197:0: C0301: Line too long (101/100) (line-too-long)
compile_phase.py:204:0: C0301: Line too long (110/100) (line-too-long)
compile_phase.py:206:0: C0301: Line too long (112/100) (line-too-long)
compile_phase.py:209:0: C0301: Line too long (111/100) (line-too-long)
compile_phase.py:210:0: C0301: Line too long (101/100) (line-too-long)
compile_phase.py:227:0: C0301: Line too long (118/100) (line-too-long)
compile_phase.py:234:0: C0301: Line too long (111/100) (line-too-long)
compile_phase.py:239:0: C0301: Line too long (106/100) (line-too-long)
compile_phase.py:246:0: C0301: Line too long (109/100) (line-too-long)
compile_phase.py:247:0: C0301: Line too long (102/100) (line-too-long)
compile_phase.py:252:0: C0301: Line too long (116/100) (line-too-long)
compile_phase.py:278:0: C0301: Line too long (113/100) (line-too-long)
compile_phase.py:281:0: C0301: Line too long (112/100) (line-too-long)
compile_phase.py:284:0: C0301: Line too long (117/100) (line-too-long)
compile_phase.py:287:0: C0301: Line too long (113/100) (line-too-long)
compile_phase.py:297:0: C0301: Line too long (107/100) (line-too-long)
compile_phase.py:298:0: C0301: Line too long (104/100) (line-too-long)
compile_phase.py:299:0: C0301: Line too long (102/100) (line-too-long)
compile_phase.py:300:0: C0301: Line too long (104/100) (line-too-long)
compile_phase.py:301:0: C0301: Line too long (116/100) (line-too-long)
compile_phase.py:302:0: C0301: Line too long (101/100) (line-too-long)
compile_phase.py:305:0: C0301: Line too long (112/100) (line-too-long)
compile_phase.py:311:0: C0301: Line too long (120/100) (line-too-long)
compile_phase.py:315:0: C0301: Line too long (115/100) (line-too-long)
compile_phase.py:318:0: C0301: Line too long (117/100) (line-too-long)
compile_phase.py:321:0: C0301: Line too long (110/100) (line-too-long)
compile_phase.py:323:0: C0301: Line too long (113/100) (line-too-long)
compile_phase.py:328:0: C0301: Line too long (114/100) (line-too-long)
compile_phase.py:331:0: C0301: Line too long (104/100) (line-too-long)
compile_phase.py:332:0: C0301: Line too long (114/100) (line-too-long)
compile_phase.py:333:0: C0301: Line too long (116/100) (line-too-long)
compile_phase.py:334:0: C0301: Line too long (101/100) (line-too-long)
compile_phase.py:336:0: C0301: Line too long (111/100) (line-too-long)
compile_phase.py:337:0: C0301: Line too long (112/100) (line-too-long)
compile_phase.py:342:0: C0301: Line too long (110/100) (line-too-long)
compile_phase.py:348:0: C0301: Line too long (117/100) (line-too-long)
compile_phase.py:351:0: C0301: Line too long (115/100) (line-too-long)
compile_phase.py:381:0: C0301: Line too long (105/100) (line-too-long)
compile_phase.py:411:0: C0301: Line too long (118/100) (line-too-long)
compile_phase.py:412:0: C0301: Line too long (118/100) (line-too-long)
compile_phase.py:415:0: C0301: Line too long (101/100) (line-too-long)
compile_phase.py:416:0: C0301: Line too long (107/100) (line-too-long)
compile_phase.py:441:0: C0301: Line too long (108/100) (line-too-long)
```

```
compile_phase.py:445:0: C0301: Line too long (115/100) (line-too-long)
compile_phase.py:448:0: C0301: Line too long (101/100) (line-too-long)
compile_phase.py:449:0: C0301: Line too long (107/100) (line-too-long)
compile_phase.py:101:2: W0511: TODO Does it always end like this?! How about "omitted n warnings"? (fixme)
compile_phase.py:17:0: C0116: Missing function or method docstring (missing-function-docstring)
compile_phase.py:23:0: C0116: Missing function or method docstring (missing-function-docstring)
compile_phase.py:67:4: C0103: Variable name "e" doesn't conform to snake_case naming style (invalid-name)
compile_phase.py:72:0: C0116: Missing function or method docstring (missing-function-docstring)
compile_phase.py:110:0: C0116: Missing function or method docstring (missing-function-docstring)
compile_phase.py:161:0: C0116: Missing function or method docstring (missing-function-docstring)
compile_phase.py:193:0: C0116: Missing function or method docstring (missing-function-docstring)
compile_phase.py:197:0: R0913: Too many arguments (8/5) (too-many-arguments)
compile_phase.py:229:4: C0103: Variable name "e" doesn't conform to snake_case naming style (invalid-name)
compile_phase.py:246:0: C0116: Missing function or method docstring (missing-function-docstring)
compile_phase.py:246:0: R0913: Too many arguments (6/5) (too-many-arguments)
compile_phase.py:258:29: R1719: The if expression can be replaced with 'bool(test)' (simplifiable-if-expression)
compile_phase.py:259:12: C0103: Variable name "r" doesn't conform to snake_case naming style (invalid-name)
compile_phase.py:266:12: W1510: Using subprocess.run without explicitly set `check` is not recommended.
(subprocess-run-check)
compile_phase.py:311:0: R0913: Too many arguments (6/5) (too-many-arguments)
compile_phase.py:342:0: R0913: Too many arguments (6/5) (too-many-arguments)
compile_phase.py:368:4: C0103: Variable name "e" doesn't conform to snake_case naming style (invalid-name)
compile_phase.py:388:39: C0321: More than one statement on a single line (multiple-statements)
compile_phase.py:413:4: C0103: Variable name "e" doesn't conform to snake_case naming style (invalid-name)
compile_phase.py:446:4: C0103: Variable name "e" doesn't conform to snake_case naming style (invalid-name)
************* Module execution_phase
execution_phase.py:2:0: C0301: Line too long (117/100) (line-too-long)
execution_phase.py:19:0: C0301: Line too long (119/100) (line-too-long)
execution_phase.py:99:0: C0301: Line too long (101/100) (line-too-long)
execution_phase.py:117:0: C0301: Line too long (101/100) (line-too-long)
execution_phase.py:13:0: C0115: Missing class docstring (missing-class-docstring)
execution_phase.py:53:0: C0116: Missing function or method docstring (missing-function-docstring)
execution_phase.py:61:0: C0116: Missing function or method docstring (missing-function-docstring)
execution_phase.py:71:0: C0116: Missing function or method docstring (missing-function-docstring)
execution_phase.py:94:17: W1510: Using subprocess.run without explicitly set `check` is not recommended.
(subprocess-run-check)
execution_phase.py:96:4: C0103: Variable name "e1" doesn't conform to snake_case naming style (invalid-name)
execution_phase.py:99:25: W1510: Using subprocess.run without explicitly set `check` is not recommended.
(subprocess-run-check)
execution_phase.py:101:12: C0103: Variable name "e2" doesn't conform to snake_case naming style (invalid-name)
************* Module output_classes
output_classes.py:2:0: C0301: Line too long (116/100) (line-too-long)
output_classes.py:15:0: C0301: Line too long (109/100) (line-too-long)
output_classes.py:30:0: C0301: Line too long (118/100) (line-too-long)
output_classes.py:86:0: C0301: Line too long (119/100) (line-too-long)
output_classes.py:89:0: C0301: Line too long (103/100) (line-too-long)
output_classes.py:93:0: C0301: Line too long (105/100) (line-too-long)
output_classes.py:98:0: C0301: Line too long (103/100) (line-too-long)
output_classes.py:99:0: C0301: Line too long (101/100) (line-too-long)
output_classes.py:103:0: C0301: Line too long (103/100) (line-too-long)
output_classes.py:104:0: C0301: Line too long (101/100) (line-too-long)
output_classes.py:108:0: C0301: Line too long (115/100) (line-too-long)
output_classes.py:119:0: C0301: Line too long (109/100) (line-too-long)
output_classes.py:140:0: C0301: Line too long (128/100) (line-too-long)
output_classes.py:144:0: C0301: Line too long (117/100) (line-too-long)
output_classes.py:45:-1: W0105: String statement has no effect (pointless-string-statement)
output_classes.py:76:4: C0116: Missing function or method docstring (missing-function-docstring)
output_classes.py:81:4: C0116: Missing function or method docstring (missing-function-docstring)
output_classes.py:131:4: C0116: Missing function or method docstring (missing-function-docstring)
output_classes.py:136:4: C0116: Missing function or method docstring (missing-function-docstring)
************* Module recalculate_scores_from_table
recalculate_scores_from_table.py:18:0: C0301: Line too long (101/100) (line-too-long)
recalculate_scores_from_table.py:66:0: C0301: Line too long (175/100) (line-too-long)
recalculate_scores_from_table.py:67:0: C0301: Line too long (164/100) (line-too-long)
recalculate_scores_from_table.py:77:0: C0301: Line too long (108/100) (line-too-long)
recalculate_scores_from_table.py:103:0: C0301: Line too long (114/100) (line-too-long)
recalculate_scores_from_table.py:111:0: C0301: Line too long (110/100) (line-too-long)
recalculate_scores_from_table.py:119:0: C0301: Line too long (116/100) (line-too-long)
```

```
recalculate_scores_from_table.py:126:0: C0301: Line too long (108/100) (line-too-long)
recalculate_scores_from_table.py:135:0: C0301: Line too long (103/100) (line-too-long)
recalculate_scores_from_table.py:136:0: C0301: Line too long (109/100) (line-too-long)
recalculate_scores_from_table.py:145:0: C0301: Line too long (104/100) (line-too-long)
recalculate_scores_from_table.py:153:0: C0301: Line too long (107/100) (line-too-long)
recalculate_scores_from_table.py:161:0: C0301: Line too long (101/100) (line-too-long)
recalculate_scores_from_table.py:169:0: C0301: Line too long (115/100) (line-too-long)
recalculate_scores_from_table.py:174:0: C0301: Line too long (102/100) (line-too-long)
recalculate_scores_from_table.py:176:0: C0301: Line too long (120/100) (line-too-long)
recalculate_scores_from_table.py:225:0: C0301: Line too long (118/100) (line-too-long)
recalculate_scores_from_table.py:87:2: W0511: TODO: fix this, this was used to add a new category to the table (fixme)
recalculate_scores_from_table.py:98:2: W0511: TODO: turn this pile of shame into elegant code someday (fixme)
recalculate_scores_from_table.py:15:0: C0116: Missing function or method docstring (missing-function-docstring)
recalculate_scores_from_table.py:15:0: R0914: Too many local variables (56/15) (too-many-locals)
recalculate_scores_from_table.py:60:4: C0103: Variable name "d" doesn't conform to snake_case naming style (invalid-name)
recalculate_scores_from_table.py:74:31: C0321: More than one statement on a single line (multiple-statements)
recalculate_scores_from_table.py:78:43: C0321: More than one statement on a single line (multiple-statements)
recalculate_scores_from_table.py:79:27: C0321: More than one statement on a single line (multiple-statements)
recalculate_scores_from_table.py:89:33: C0321: More than one statement on a single line (multiple-statements)
recalculate_scores_from_table.py:91:46: C0321: More than one statement on a single line (multiple-statements)
recalculate_scores_from_table.py:93:42: C0321: More than one statement on a single line (multiple-statements)
recalculate_scores_from_table.py:220:33: C0321: More than one statement on a single line (multiple-statements)
recalculate_scores_from_table.py:238:4: C0103: Variable name "n" doesn't conform to snake_case naming style (invalid-name)
recalculate_scores_from_table.py:239:4: C0103: Variable name "rc" doesn't conform to snake_case naming style (invalid-name)
recalculate_scores_from_table.py:15:0: R0912: Too many branches (28/12) (too-many-branches)
recalculate_scores_from_table.py:15:0: R0915: Too many statements (171/50) (too-many-statements)
************* Module scoring
scoring.py:34:0: C0301: Line too long (112/100) (line-too-long)
scoring.py:35:0: C0301: Line too long (117/100) (line-too-long)
scoring.py:42:0: C0301: Line too long (115/100) (line-too-long)
scoring.py:43:0: C0301: Line too long (117/100) (line-too-long)
scoring.py:46:0: C0301: Line too long (117/100) (line-too-long)
scoring.py:65:0: C0301: Line too long (114/100) (line-too-long)
scoring.py:66:0: C0301: Line too long (118/100) (line-too-long)
scoring.py:67:0: C0301: Line too long (117/100) (line-too-long)
scoring.py:68:0: C0301: Line too long (117/100) (line-too-long)
scoring.py:69:0: C0301: Line too long (117/100) (line-too-long)
scoring.py:70:0: C0301: Line too long (110/100) (line-too-long)
scoring.py:72:0: C0301: Line too long (115/100) (line-too-long)
scoring.py:73:0: C0301: Line too long (117/100) (line-too-long)
scoring.py:138:0: C0301: Line too long (117/100) (line-too-long)
scoring.py:139:0: C0301: Line too long (121/100) (line-too-long)
scoring.py:400:0: C0301: Line too long (110/100) (line-too-long)
scoring.py:404:0: C0301: Line too long (107/100) (line-too-long)
scoring.py:18:0: C0116: Missing function or method docstring (missing-function-docstring)
scoring.py:85:4: C0103: Variable name "d" doesn't conform to snake_case naming style (invalid-name)
scoring.py:86:4: C0103: Variable name "x" doesn't conform to snake_case naming style (invalid-name)
scoring.py:92:12: C0103: Variable name "x0" doesn't conform to snake_case naming style (invalid-name)
scoring.py:99:12: C0103: Variable name "x0" doesn't conform to snake_case naming style (invalid-name)
scoring.py:105:12: C0103: Variable name "a" doesn't conform to snake_case naming style (invalid-name)
scoring.py:106:12: C0103: Variable name "b" doesn't conform to snake_case naming style (invalid-name)
scoring.py:108:12: C0103: Variable name "a" doesn't conform to snake_case naming style (invalid-name)
scoring.py:109:12: C0103: Variable name "b" doesn't conform to snake_case naming style (invalid-name)
scoring.py:111:12: C0103: Variable name "a" doesn't conform to snake_case naming style (invalid-name)
scoring.py:112:12: C0103: Variable name "b" doesn't conform to snake_case naming style (invalid-name)
scoring.py:126:4: C0103: Variable name "d" doesn't conform to snake_case naming style (invalid-name)
scoring.py:127:4: C0103: Variable name "x" doesn't conform to snake_case naming style (invalid-name)
scoring.py:131:10: W0612: Unused variable 'pcov' (unused-variable)
scoring.py:149:4: C0103: Variable name "d" doesn't conform to snake_case naming style (invalid-name)
scoring.py:150:4: C0103: Variable name "x" doesn't conform to snake_case naming style (invalid-name)
scoring.py:171:0: C0103: Argument name "x" doesn't conform to snake_case naming style (invalid-name)
scoring.py:171:0: C0103: Argument name "x0" doesn't conform to snake_case naming style (invalid-name)
scoring.py:175:4: C0103: Variable name "y" doesn't conform to snake_case naming style (invalid-name)
scoring.py:209:-1: W0105: String statement has no effect (pointless-string-statement)
scoring.py:339:0: C0116: Missing function or method docstring (missing-function-docstring)
scoring.py:343:0: C0116: Missing function or method docstring (missing-function-docstring)
scoring.py:347:0: C0116: Missing function or method docstring (missing-function-docstring)
scoring.py:351:0: C0116: Missing function or method docstring (missing-function-docstring)
```

```
scoring.py:355:0: C0116: Missing function or method docstring (missing-function-docstring)
scoring.py:359:0: C0116: Missing function or method docstring (missing-function-docstring)
scoring.py:363:0: C0116: Missing function or method docstring (missing-function-docstring)
scoring.py:367:0: C0116: Missing function or method docstring (missing-function-docstring)
scoring.py:371:0: C0116: Missing function or method docstring (missing-function-docstring)
scoring.py:375:0: C0116: Missing function or method docstring (missing-function-docstring)
scoring.py:379:0: C0116: Missing function or method docstring (missing-function-docstring)
scoring.py:383:0: C0116: Missing function or method docstring (missing-function-docstring)
scoring.py:387:0: C0116: Missing function or method docstring (missing-function-docstring)
scoring.py:391:0: C0116: Missing function or method docstring (missing-function-docstring)
scoring.py:395:0: C0116: Missing function or method docstring (missing-function-docstring)
scoring.py:399:0: C0116: Missing function or method docstring (missing-function-docstring)
scoring.py:403:0: C0116: Missing function or method docstring (missing-function-docstring)
scoring.py:407:0: C0116: Missing function or method docstring (missing-function-docstring)
scoring.py:411:0: C0116: Missing function or method docstring (missing-function-docstring)
scoring.py:415:0: C0116: Missing function or method docstring (missing-function-docstring)
scoring.py:419:0: C0116: Missing function or method docstring (missing-function-docstring)
scoring.py:423:0: C0116: Missing function or method docstring (missing-function-docstring)
************* Module setup
setup.py:20:0: C0304: Final newline missing (missing-final-newline)
setup.py:5:0: W0611: Unused find_packages imported from setuptools (unused-import)
************* Module softwipe
softwipe.py:18:0: C0301: Line too long (119/100) (line-too-long)
softwipe.py:27:0: C0301: Line too long (117/100) (line-too-long)
softwipe.py:28:0: C0301: Line too long (110/100) (line-too-long)
softwipe.py:50:0: C0301: Line too long (104/100) (line-too-long)
softwipe.py:51:0: C0301: Line too long (113/100) (line-too-long)
softwipe.py:53:0: C0301: Line too long (117/100) (line-too-long)
softwipe.py:54:0: C0301: Line too long (104/100) (line-too-long)
softwipe.py:55:0: C0301: Line too long (110/100) (line-too-long)
softwipe.py:65:0: C0301: Line too long (120/100) (line-too-long)
softwipe.py:67:0: C0301: Line too long (113/100) (line-too-long)
softwipe.py:68:0: C0301: Line too long (115/100) (line-too-long)
softwipe.py:69:0: C0301: Line too long (116/100) (line-too-long)
softwipe.py:71:0: C0301: Line too long (119/100) (line-too-long)
softwipe.py:72:0: C0301: Line too long (119/100) (line-too-long)
softwipe.py:75:0: C0301: Line too long (119/100) (line-too-long)
softwipe.py:77:0: C0301: Line too long (115/100) (line-too-long)
softwipe.py:78:0: C0301: Line too long (101/100) (line-too-long)
softwipe.py:80:0: C0301: Line too long (117/100) (line-too-long)
softwipe.py:81:0: C0301: Line too long (111/100) (line-too-long)
softwipe.py:83:0: C0301: Line too long (114/100) (line-too-long)
softwipe.py:84:0: C0301: Line too long (101/100) (line-too-long)
softwipe.py:86:0: C0301: Line too long (111/100) (line-too-long)
softwipe.py:87:0: C0301: Line too long (115/100) (line-too-long)
softwipe.py:88:0: C0301: Line too long (118/100) (line-too-long)
softwipe.py:90:0: C0301: Line too long (118/100) (line-too-long)
softwipe.py:91:0: C0301: Line too long (117/100) (line-too-long)
softwipe.py:94:0: C0301: Line too long (118/100) (line-too-long)
softwipe.py:95:0: C0301: Line too long (115/100) (line-too-long)
softwipe.py:96:0: C0301: Line too long (114/100) (line-too-long)
softwipe.py:99:0: C0301: Line too long (115/100) (line-too-long)
softwipe.py:100:0: C0301: Line too long (117/100) (line-too-long)
softwipe.py:101:0: C0301: Line too long (107/100) (line-too-long)
softwipe.py:103:0: C0301: Line too long (114/100) (line-too-long)
softwipe.py:106:0: C0301: Line too long (113/100) (line-too-long)
softwipe.py:107:0: C0301: Line too long (115/100) (line-too-long)
softwipe.py:108:0: C0301: Line too long (116/100) (line-too-long)
softwipe.py:109:0: C0301: Line too long (108/100) (line-too-long)
softwipe.py:111:0: C0301: Line too long (117/100) (line-too-long)
softwipe.py:116:0: C0301: Line too long (112/100) (line-too-long)
softwipe.py:117:0: C0301: Line too long (106/100) (line-too-long)
softwipe.py:118:0: C0301: Line too long (110/100) (line-too-long)
softwipe.py:119:0: C0301: Line too long (105/100) (line-too-long)
softwipe.py:122:0: C0301: Line too long (106/100) (line-too-long)
softwipe.py:123:0: C0301: Line too long (110/100) (line-too-long)
softwipe.py:124:0: C0301: Line too long (108/100) (line-too-long)
softwipe.py:125:0: C0301: Line too long (114/100) (line-too-long)
```

```
softwipe.py:146:0: C0301: Line too long (117/100) (line-too-long)
softwipe.py:181:0: C0301: Line too long (110/100) (line-too-long)
softwipe.py:212:0: C0301: Line too long (117/100) (line-too-long)
softwipe.py:223:0: C0301: Line too long (118/100) (line-too-long)
softwipe.py:226:0: C0301: Line too long (118/100) (line-too-long)
softwipe.py:228:0: C0301: Line too long (111/100) (line-too-long)
softwipe.py:232:0: C0301: Line too long (119/100) (line-too-long)
softwipe.py:235:0: C0301: Line too long (119/100) (line-too-long)
softwipe.py:251:0: C0301: Line too long (109/100) (line-too-long)
softwipe.py:253:0: C0301: Line too long (108/100) (line-too-long)
softwipe.py:272:0: C0301: Line too long (112/100) (line-too-long)
softwipe.py:279:0: C0301: Line too long (106/100) (line-too-long)
softwipe.py:296:0: C0301: Line too long (113/100) (line-too-long)
softwipe.py:300:0: C0301: Line too long (118/100) (line-too-long)
softwipe.py:305:0: C0301: Line too long (116/100) (line-too-long)
softwipe.py:359:0: C0301: Line too long (107/100) (line-too-long)
softwipe.py:393:0: C0301: Line too long (111/100) (line-too-long)
softwipe.py:412:0: C0301: Line too long (107/100) (line-too-long)
softwipe.py:158:2: W0511: TODO: fix versioning (fixme)
softwipe.py:168:2: W0511: TODO: fix versioning (fixme)
softwipe.py:255:2: W0511: TODO: allow clang compilation as well!!! (fixme)
softwipe.py:313:2: W0511: TODO: Clean and test this function (fixme)
softwipe.py:356:2: W0511: TODO: hopefully get a conda package for this sometime (fixme)
softwipe.py:393:2: W0511: TODO: maybe add valgrind at some point if we get its error counts normalized somehow (fixme)
softwipe.py:412:2: W0511: TODO: maybe completely remove Infer since it requires a lot of disk space (fixme)
softwipe.py:60:4: C0103: Variable name "c" doesn’t conform to snake_case naming style (invalid-name)
softwipe.py:157:0: C0116: Missing function or method docstring (missing-function-docstring)
softwipe.py:167:0: C0116: Missing function or method docstring (missing-function-docstring)
softwipe.py:197:12: R1723: Unnecessary "elif" after "break" (no-else-break)
softwipe.py:279:0: R0913: Too many arguments (6/5) (too-many-arguments)
softwipe.py:352:0: R0914: Too many local variables (20/15) (too-many-locals)
softwipe.py:352:0: R0912: Too many branches (15/12) (too-many-branches)
softwipe.py:352:0: R0915: Too many statements (53/50) (too-many-statements)
softwipe.py:10:0: W0611: Unused ThreadPool imported from multiprocessing.pool (unused-import)
softwipe.py:18:0: W0611: Unused ClangTool imported from analysis_tools (unused-import)
softwipe.py:18:0: W0611: Unused ValgrindTool imported from analysis_tools (unused-import)
************* Module strings
strings.py:5:0: C0301: Line too long (148/100) (line-too-long)
strings.py:7:0: C0301: Line too long (115/100) (line-too-long)
strings.py:9:0: C0301: Line too long (104/100) (line-too-long)
strings.py:28:0: C0301: Line too long (114/100) (line-too-long)
strings.py:31:0: C0301: Line too long (119/100) (line-too-long)
strings.py:32:0: C0301: Line too long (114/100) (line-too-long)
strings.py:33:0: C0301: Line too long (113/100) (line-too-long)
strings.py:39:0: C0301: Line too long (110/100) (line-too-long)
strings.py:40:0: C0301: Line too long (119/100) (line-too-long)
strings.py:47:0: C0301: Line too long (104/100) (line-too-long)
strings.py:60:0: C0301: Line too long (113/100) (line-too-long)
strings.py:99:0: C0301: Line too long (147/100) (line-too-long)
strings.py:108:0: C0301: Line too long (118/100) (line-too-long)
strings.py:109:0: C0301: Line too long (115/100) (line-too-long)
strings.py:112:0: C0301: Line too long (116/100) (line-too-long)
strings.py:113:0: C0301: Line too long (112/100) (line-too-long)
strings.py:117:0: C0301: Line too long (118/100) (line-too-long)
strings.py:120:0: C0301: Line too long (120/100) (line-too-long)
strings.py:121:0: C0301: Line too long (111/100) (line-too-long)
strings.py:122:0: C0301: Line too long (120/100) (line-too-long)
strings.py:123:0: C0301: Line too long (139/100) (line-too-long)
strings.py:124:0: C0301: Line too long (145/100) (line-too-long)
strings.py:127:0: C0301: Line too long (109/100) (line-too-long)
************* Module tools_info
tools_info.py:14:0: R0903: Too few public methods (0/2) (too-few-public-methods)
************* Module util
util.py:18:0: C0301: Line too long (112/100) (line-too-long)
util.py:30:0: C0301: Line too long (121/100) (line-too-long)
util.py:55:0: C0301: Line too long (117/100) (line-too-long)
util.py:58:0: C0301: Line too long (113/100) (line-too-long)
util.py:71:0: C0301: Line too long (111/100) (line-too-long)
```

```
util.py:74:0: C0301: Line too long (108/100) (line-too-long)
util.py:101:0: C0301: Line too long (122/100) (line-too-long)
util.py:112:0: C0301: Line too long (107/100) (line-too-long)
util.py:226:0: C0304: Final newline missing (missing-final-newline)
util.py:10:0: C0116: Missing function or method docstring (missing-function-docstring)
util.py:95:0: C0116: Missing function or method docstring (missing-function-docstring)
util.py:168:0: C0116: Missing function or method docstring (missing-function-docstring)
util.py:172:0: C0116: Missing function or method docstring (missing-function-docstring)
util.py:215:0: C0116: Missing function or method docstring (missing-function-docstring)
util.py:221:0: C0116: Missing function or method docstring (missing-function-docstring)
util.py:7:0: C0411: standard import "import shutil" should be placed before "import strings" (wrong-import-order)
util.py:1:0: R0801: Similar lines in 2 files
==analysis_tools:593
==compile_phase:434
    infer_call.extend(["--", "make"])
    make_clean_call = ["make", "clean"]

    try:
        subprocess.check_output(make_clean_call, cwd=program_dir_abs, universal_newlines=True,
                                stderr=subprocess.STDOUT)
    except subprocess.CalledProcessError:  # not all makefiles have a clean option, pass if it doesn't exist
        pass

    try:
        subprocess.check_output(infer_call, cwd=program_dir_abs, universal_newlines=True, stderr=subprocess.STDOUT)
    except subprocess.CalledProcessError as e:
        util.write_into_file_string(strings.ERROR_FILENAME_INFER_COMPILATION,
                                    strings.INFER_COMPILATION_CRASHED.format(e.returncode, e.output))
        print(strings.INFER_COMPILATION_CRASHED.format(e.returncode, strings.ERROR_LOG_WRITTEN_INTO.format(
            strings.ERROR_FILENAME_INFER_COMPILATION)))
        print()
        return False (duplicate-code)
util.py:1:0: R0801: Similar lines in 2 files
==compile_phase:16
==util:214
def create_build_directory(program_dir_abs, build_dir_name=strings.SOFTWIPE_BUILD_DIR_NAME):
    build_path = os.path.join(program_dir_abs, build_dir_name)
    os.makedirs(build_path, exist_ok=True)
    return build_path


def clear_directory(directory):
    for path in (os.path.join(directory, file) for file in os.listdir(directory)):
        if os.path.isdir(path):
            shutil.rmtree(path)
        else:
            os.remove(path) (duplicate-code)
util.py:1:0: R0801: Similar lines in 2 files
==compare_results:45
==recalculate_scores_from_table:46
    rates = {
        'compiler_and_sanitizer': [],
        'assertions': [],
        'cppcheck': [],
        'clang_tidy': [],
        'cyclomatic_complexity': [],
        'lizard_warnings': [],
        'unique': [],
        'kwstyle': [], (duplicate-code)
util.py:1:0: R0801: Similar lines in 3 files
==analysis_tools:573
==compile_phase:413
==compile_phase:446
            util.write_into_file_string(strings.ERROR_FILENAME_INFER_COMPILATION,
                                        strings.INFER_COMPILATION_CRASHED.format(e.returncode, e.output))
            print(strings.INFER_COMPILATION_CRASHED.format(e.returncode, strings.ERROR_LOG_WRITTEN_INTO.format(
                strings.ERROR_FILENAME_INFER_COMPILATION)))
            print()
```

```
            return False (duplicate-code)
util.py:1:0: R0801: Similar lines in 2 files
==analysis_tools:79
==softwipe:226
        elif args.clang:
            score = compile_phase.compile_program_clang(program_dir_abs, args.clang, lines_of_code, compiler_flags,
                                                        excluded_paths, cpp)
        else:
            if command_file: (duplicate-code)

------------------------------------------------------------------
Your code has been rated at 7.17/10 (previous run: 7.14/10, +0.02)
```

6.4 Lizard Output

The output of Lizard is also available at
https://github.com/adrianzap/softwipe/blob/master/softwipe_code_quality/lizard_results.txt.

```
    ================================================
  NLOC    CCN   token  PARAM  length  location
------------------------------------------------
       2      1      6      1       2 __init__@24-25@./analysis_tools.py
       2      1     17      2       8 run@28-35@./analysis_tools.py
       2      1      6      0       2 name@38-39@./analysis_tools.py
       2      1     10      2       2 run@44-45@./analysis_tools.py
       2      1      6      0       2 name@48-49@./analysis_tools.py
      36      5    159      3      38 run@55-92@./analysis_tools.py
       2      1      6      0       2 name@95-96@./analysis_tools.py
       8      3     56      2      19 is_assert@101-119@./analysis_tools.py
      23      4    183      2      32 run@122-153@./analysis_tools.py
       2      1      6      0       2 name@156-157@./analysis_tools.py
      12      5     59      1      21 get_warning_lines@162-182@./analysis_tools.py
      11      4     73      1      21 get_weighted_warning_count@185-205@./analysis_tools.py
       6      4     41      1      13 beatify_warning_lines@208-220@./analysis_tools.py
      45      7    268      3      57 run@223-279@./analysis_tools.py
       2      1      6      0       2 name@282-283@./analysis_tools.py
      25      7    160      1      40 filter_output@288-327@./analysis_tools.py
      25      4    157      2      36 run@330-365@./analysis_tools.py
       2      1      6      0       2 name@368-369@./analysis_tools.py
       7      3     35      1      11 get_warning_lines@374-384@./analysis_tools.py
      35      7    258      2      43 run@387-429@./analysis_tools.py
       2      1      6      0       2 name@432-433@./analysis_tools.py
       7      3     35      1      14 get_warning_count@438-451@./analysis_tools.py
      27      5    221      2      38 run@454-491@./analysis_tools.py
       2      1      6      0       2 name@494-495@./analysis_tools.py
      24      7    126      1      34 get_warnings_from_output@500-533@./analysis_tools.py
       8      3     51      2      14 prepare_exclude_arguments@536-549@./analysis_tools.py
      20      2    172      2      29 compile_with_cmake@552-580@./analysis_tools.py
      21      3    163      2      30 compile_with_make@583-612@./analysis_tools.py
      44      9    320      2      55 run@615-669@./analysis_tools.py
       2      1      6      0       2 name@672-673@./analysis_tools.py
       7      4     35      1       7 get_weighted_warning_count@678-684@./analysis_tools.py
      11      5     56      1      11 get_warning_log@687-697@./analysis_tools.py
      43      7    318      2      56 run@700-755@./analysis_tools.py
       2      1      6      0       2 name@758-759@./analysis_tools.py
      12      3    116      2      16 run@764-779@./analysis_tools.py
       2      1      6      0       2 name@782-783@./analysis_tools.py
       9      2     42      0      13 detect_user_os@18-30@./automatic_tool_installation.py
       7      3     44      1       7 get_package_install_command_for_os@33-39@./automatic_tool_installation.py
       5      2     44      1       6 print_missing_tools@42-47@./automatic_tool_installation.py
       6      2     30      1       6 print_and_run_install_command@50-55@./automatic_tool_installation.py
      13      4     66      0      16 handle_libtinfo_download@58-73@./automatic_tool_installation.py
       7      3     45      1       7 install_apt_package_if_needed@76-82@./automatic_tool_installation.py
      12      1     81      0      14 handle_kwstyle_download@85-98@./automatic_tool_installation.py
       9      1     85      0       9 handle_lizard_download@101-109@./automatic_tool_installation.py
```

8	1	77	0	9	handle_infer_download@112-120@./automatic_tool_installation.py
7	4	30	1	11	handle_tool_download@123-133@./automatic_tool_installation.py
5	2	30	1	10	handle_clang_tidy_installation@136-145@./automatic_tool_installation.py
15	6	102	2	18	auto_tool_install@148-165@./automatic_tool_installation.py
11	4	62	2	11	auto_install_prompt@168-178@./automatic_tool_installation.py
15	8	126	0	24	check_if_all_required_tools_are_installed@181-204@./automatic_tool_installation.py
15	1	78	0	17	parse_arguments@56-72@./calculate_score_table.py
49	26	467	2	61	get_result_rates@75-135@./calculate_score_table.py
2	1	22	1	2	get_result_values.get_absolute_value@139-140@./calculate_score_table.py
53	28	533	2	62	get_result_values@138-199@./calculate_score_table.py
87	21	629	2	104	calculate_scores@202-305@./calculate_score_table.py
34	11	313	4	38	print_score_csv@308-345@./calculate_score_table.py
7	3	66	0	7	main@348-354@./calculate_score_table.py
24	1	156	0	26	parse_arguments@16-41@./compare_results.py
25	2	206	1	30	get_all_rates@44-73@./compare_results.py
6	3	59	1	12	sort_rates@76-87@./compare_results.py
9	2	76	1	9	calculate_median@90-98@./compare_results.py
9	6	72	3	11	print_average_and_median@101-111@./compare_results.py
11	5	83	1	15	get_turkeys_fences@114-128@./compare_results.py
14	5	101	3	16	print_all_rates@131-146@./compare_results.py
44	15	231	1	49	print_softwipe_scoring_values@149-197@./compare_results.py
11	3	145	4	11	print_best_rates@200-210@./compare_results.py
2	1	25	3	2	print_best_rates_only@213-214@./compare_results.py
13	4	86	0	15	main@217-231@./compare_results.py
4	1	35	2	4	create_build_directory@17-20@./compile_phase.py
6	4	54	1	6	clear_directory@23-28@./compile_phase.py
9	1	69	2	23	build_cmake_call@31-53@./compile_phase.py
7	2	72	3	14	run_cmake@56-69@./compile_phase.py
3	1	18	1	5	line_is_warning_line@72-76@./compile_phase.py
12	5	61	1	29	get_warning_lines_from_make_output@79-107@./compile_phase.py
38	7	242	3	49	print_compilation_results@110-158@./compile_phase.py
22	10	129	2	30	remove_excluded_paths_from_warning_lines@161-190@./compile_phase.py
2	1	16	1	2	running_make_clean@193-194@./compile_phase.py
2	1	29	8	2	run_make@197-198@./compile_phase.py
2	1	15	6	2	parse_make_command_file_and_run_all_commands_in_it@246-247@./compile_phase.py
17	3	92	5	31	compile_program_make@278-308@./compile_phase.py
2	1	19	6	2	compile_program_cmake@311-312@./compile_phase.py
18	4	162	6	35	compile_program_clang@342-376@./compile_phase.py
7	3	51	2	13	get_infer_exclude_arguments@379-391@./compile_phase.py
18	2	166	2	28	compile_program_infer_cmake@394-421@./compile_phase.py
21	3	157	2	31	compile_program_infer_make@424-454@./compile_phase.py
21	5	153	3	34	build_command@17-50@./execution_phase.py
6	4	30	1	6	get_asan_error_count_from_sanitizer_output_lines@53-58@./execution_phase.py
7	3	32	1	8	get_ubsan_error_count_from_sanitizer_output_lines@61-68@./execution_phase.py
5	1	29	1	5	get_sanitizer_error_count_from_sanitizer_output@71-75@./execution_phase.py
30	4	239	4	44	run_execution@78-121@./execution_phase.py
52	10	165	2	56	__init__@19-74@./output_classes.py
4	1	23	2	4	print_information@76-79@./output_classes.py
27	6	209	2	30	get_information@81-110@./output_classes.py
5	1	33	5	5	__init__@125-129@./output_classes.py
4	1	28	1	4	print_information_and_return_scores@131-134@./output_classes.py
14	1	135	1	18	get_information@136-153@./output_classes.py
192	35	1760	0	233	main@15-247@./recalculate_scores_from_table.py
3	1	22	2	8	print_score@8-15@./scoring.py
2	1	23	2	2	get_score_string@18-19@./scoring.py
3	1	22	1	8	average_score@22-29@./scoring.py
7	3	53	3	22	_calculate_score_generic@32-53@./scoring.py
2	1	27	4	5	_calculate_score_absolute@56-60@./scoring.py
30	7	244	4	52	_calculate_score_smooth_linear@63-114@./scoring.py
8	1	103	3	17	_calculate_score_curve_fit@117-133@./scoring.py
21	8	134	4	34	_calculate_score_curve_fit_combined@136-169@./scoring.py
3	1	33	3	6	sigmoid@171-176@./scoring.py
2	1	14	1	2	calculate_compiler_and_sanitizer_score@339-340@./scoring.py
2	1	14	1	2	calculate_assertion_score@343-344@./scoring.py
2	1	14	1	2	calculate_cppcheck_score@347-348@./scoring.py
2	1	14	1	2	calculate_clang_tidy_score@351-352@./scoring.py
2	1	14	1	2	calculate_cyclomatic_complexity_score@355-356@./scoring.py
2	1	14	1	2	calculate_lizard_warning_score@359-360@./scoring.py

```
       2      1     14      1       2 calculate_unique_score@363-364@./scoring.py
       2      1     14      1       2 calculate_kwstyle_score@367-368@./scoring.py
       2      1     14      1       2 calculate_infer_score@371-372@./scoring.py
       2      1     14      1       2 calculate_valgrind_score@375-376@./scoring.py
       2      1     14      1       2 calculate_testcount_score@379-380@./scoring.py
       2      1     18      1       2 calculate_compiler_and_sanitizer_score_absolute@383-384@./scoring.py
       2      1     18      1       2 calculate_assertion_score_absolute@387-388@./scoring.py
       2      1     18      1       2 calculate_cppcheck_score_absolute@391-392@./scoring.py
       2      1     18      1       2 calculate_clang_tidy_score_absolute@395-396@./scoring.py
       2      1     14      1       2 calculate_cyclomatic_complexity_score_absolute@399-400@./scoring.py
       2      1     18      1       2 calculate_lizard_warning_score_absolute@403-404@./scoring.py
       2      1     14      1       2 calculate_unique_score_absolute@407-408@./scoring.py
       2      1     18      1       2 calculate_kwstyle_score_absolute@411-412@./scoring.py
       2      1     18      1       2 calculate_infer_score_absolute@415-416@./scoring.py
       2      1     18      1       2 calculate_valgrind_score_absolute@419-420@./scoring.py
       2      1     18      1       2 calculate_testcount_score_absolute@423-424@./scoring.py
      80      7    559      0     107 parse_arguments@22-128@./softwipe.py
       6      3     43      1      11 add_to_path_variable@131-141@./softwipe.py
       6      2     53      0      10 add_kwstyle_to_path_variable@144-153@./softwipe.py
       6      2     41      0       7 add_lizard_to_path_variable@157-163@./softwipe.py
       8      2     59      0       9 add_infer_to_path_variable@167-175@./softwipe.py
       4      3     27      1       8 add_user_paths_to_path_variable@179-186@./softwipe.py
      12      5     64      0      15 warn_if_user_is_root@189-203@./softwipe.py
      20      5    151      5      32 compile_program@206-237@./softwipe.py
      10      3     61      2      20 compile_program_with_infer@240-259@./softwipe.py
       7      2     44      4      15 execute_program@262-276@./softwipe.py
       2      1     17      6       2 compile_and_execute_program_with_sanitizers@279-280@./softwipe.py
      28     11    167      2      38 add_badge_to_file@312-349@./softwipe.py
      66     17    419      0      91 main@352-442@./softwipe.py
       8      1     66      1      15 create_make_flags@14-28@./strings.py
       4      2     18      1       4 is_testfile@10-13@./util.py
       4      2     35      3      10 write_into_file_string@16-25@./util.py
       6      2     34      3      12 write_into_file_list@28-39@./util.py
       3      2     15      1       7 print_lines@42-48@./util.py
       9      3     98      2      16 get_excluded_paths@51-66@./util.py
      13      6    101      2      24 find_all_source_files@69-92@./util.py
       2      1     13      1       2 line_is_empty@95-96@./util.py
      12      7     77      2      26 line_is_comment@99-124@./util.py
      10      4     57      1      17 count_lines_of_code_in_one_file@127-143@./util.py
       5      2     22      1      12 count_lines_of_code@146-157@./util.py
       2      1     21      0       6 get_softwipe_directory@160-165@./util.py
       2      1     16      1       2 clang_tidy_output_line_is_header@168-169@./util.py
       2      1     12      1       2 clang_tidy_output_line_is_trailer@172-173@./util.py
       3      2     32      2       9 split_in_chunks@176-184@./util.py
      16      8    111      3      26 find_file@187-212@./util.py
       4      1     35      2       4 create_build_directory@215-218@./util.py
       6      4     54      1       6 clear_directory@221-226@./util.py
15 file analyzed.
==============================================================
NLOC    Avg.NLOC  AvgCCN  Avg.token  function_cnt    file
--------------------------------------------------------------
    548      13.5     3.3       87.8        36     ./analysis_tools.py
    142       9.2     3.1       61.7        14     ./automatic_tool_installation.py
    287      35.3    13.0      301.1         7     ./calculate_score_table.py
    160       0.0     0.0        0.0         0     ./classifications.py
    180      15.3     4.3      112.7        11     ./compare_results.py
    261      11.2     2.9       81.6        17     ./compile_phase.py
     79      13.8     3.4       96.6         5     ./execution_phase.py
    131      17.7     3.3       98.8         6     ./output_classes.py
    202     192.0    35.0     1760.0         1     ./recalculate_scores_from_table.py
    256       4.0     1.5       32.4        31     ./scoring.py
     18       0.0     0.0        0.0         0     ./setup.py
    288      19.6     4.8      131.2        13     ./softwipe.py
    158       8.0     1.0       66.0         1     ./strings.py
     21       0.0     0.0        0.0         0     ./tools_info.py
    109       6.1     2.9       44.2        17     ./util.py

===============================================================================================================
```

```
!!!! Warnings (cyclomatic_complexity > 15 or length > 1000 or nloc > 1000000 or parameter_count > 100) !!!!
================================================
  NLOC    CCN   token  PARAM  length  location
------------------------------------------------
      49     26    467      2      61 get_result_rates@75-135@./calculate_score_table.py
      53     28    533      2      62 get_result_values@138-199@./calculate_score_table.py
      87     21    629      2     104 calculate_scores@202-305@./calculate_score_table.py
     192     35   1760      0     233 main@15-247@./recalculate_scores_from_table.py
      66     17    419      0      91 main@352-442@./softwipe.py
==========================================================================================
Total nloc   Avg.NLOC  AvgCCN  Avg.token   Fun Cnt  Warning cnt   Fun Rt   nloc Rt
------------------------------------------------------------------------------------------
      2840      13.1     3.6       95.1      159            5      0.03    0.22
```
